# Who Has Used Internal Company Documents for Biomedical and Public Health Research and Where Did They Find Them?

**DOI:** 10.1371/journal.pone.0094709

**Published:** 2014-05-06

**Authors:** L. Susan Wieland, Lainie Rutkow, S. Swaroop Vedula, Christopher N. Kaufmann, Lori M. Rosman, Claire Twose, Nirosha Mahendraratnam, Kay Dickersin

**Affiliations:** 1 Center for Evidence-Based Medicine, Brown University, Providence, Rhode Island, United States of America; 2 Johns Hopkins Bloomberg School of Public Health, Baltimore, Maryland, United States of America; 3 Johns Hopkins University, Baltimore, Maryland, United States of America; 4 William H. Welch Medical Library, Johns Hopkins University, Baltimore, Maryland, United States of America; University of Chicago, United States of America

## Abstract

**Objective:**

To describe the sources of internal company documents used in public health and healthcare research.

**Methods:**

We searched PubMed and Embase for articles using internal company documents to address a research question about a health-related topic. Our primary interest was where authors obtained internal company documents for their research. We also extracted information on type of company, type of research question, type of internal documents, and funding source.

**Results:**

Our searches identified 9,305 citations of which 357 were eligible. Scanning of reference lists and consultation with colleagues identified 4 additional articles, resulting in 361 included articles. Most articles examined internal tobacco company documents (325/361; 90%). Articles using documents from pharmaceutical companies (20/361; 6%) were the next most common. Tobacco articles used documents from repositories; pharmaceutical documents were from a range of sources. Most included articles relied upon internal company documents obtained through litigation (350/361; 97%). The research questions posed were primarily about company strategies to promote or position the company and its products (326/361; 90%). Most articles (346/361; 96%) used information from miscellaneous documents such as memos or letters, or from unspecified types of documents. When explicit information about study funding was provided (290/361 articles), the most common source was the US-based National Cancer Institute. We developed an alternative and more sensitive search targeted at identifying additional research articles using internal pharmaceutical company documents, but the search retrieved an impractical number of citations for review.

**Conclusions:**

Internal company documents provide an excellent source of information on health topics (e.g., corporate behavior, study data) exemplified by articles based on tobacco industry documents. Pharmaceutical and other industry documents appear to have been less used for research, indicating a need for funding for this type of research and well-indexed and curated repositories to provide researchers with ready access to the documents.

## Introduction

Even though the scientific research enterprise and healthcare decisions rely on the biomedical literature being complete and accurate, it is neither [1], [2]. It is now well-established that strength and direction of findings is associated with selective reporting, and when this happens it is termed a “reporting bias” [3]. Reporting biases may manifest as omission of study findings from the literature, either completely or in part; for example, particular outcomes or analyses may be omitted [4]. Reporting biases originate mainly with the investigators, not journal editors, and occur when research is sponsored by for-profit and not-for-profit entities [5]. Funding by for-profit companies appears to be independently associated with selective reporting, however [6].

Research on selective reporting and other reporting biases is made possible when the published literature can be compared with other sources of information about the same research studies, for example from research ethics committees [7], [8], [9], funding agencies [10], [11], clinical trial registers [12], [13], documents and data released by regulatory authorities [2], [14], [15], and internal company documents released though litigation or other means [4], [16], [17], [18]. Internal company documents serve as a valuable source of information about industry-sponsored research for those producing evidence summaries [19], those concerned about an entire industry's global marketing behavior [20], and for those wishing to report a study's findings as a restorative author [21]. For example, the clinical study reports produced by pharmaceutical companies at the completion of a clinical trial typically include the protocol (what was planned) and a detailed description of study analyses and findings [17], [22]. In a study of industry trials where available internal documents were compared with publications, the primary outcome defined in the protocol (internal) disagreed with that in the publication for the majority of trials [4]. Frequently, when trials had findings that were not statistically significant (p≥0.05) for the protocol-defined primary outcome in the internal documents, they were either not published in full or were published with a changed primary outcome favoring the company's drug [17]. Examination of internal company memos and other documents indicated that alterations in what was presented in publications were part of a “publication strategy” to disseminate trial findings and influence publication content [18]. Internal documents may serve as unique sources for evidence about company activities in pursuit of strategic goals, e.g., a company's marketing or publication strategy [18], [23] and on how corporations affect health, more generally [24], [25]. Over the past 20 years, internal company documents have become increasingly available, first from tobacco companies and later from pharmaceutical companies. While the existence of tobacco company document repositories is well known, little has been published about the range of primary health research uses for internal company documents across all industries, and whether there is a need for a repository or repositories for documents from industries other than tobacco has not been addressed in the literature.

Our objective was to describe the characteristics of public health and healthcare research using internal company documents across industries. The ultimate goal of our research was to document for others the potential sources of accessible internal company data for public health and healthcare research, particularly in the area of pharmaceutical research, and, in doing so, to take the first steps toward exploring the current use and future potential for repositories of internal company information.

## Methods

### Study eligibility

Articles were eligible if they described a study that addressed a research question or objective related to public health or healthcare, and internal company documents were explicitly referred to as the source of data (i.e., information) examined in the study. We considered internal company documents to include emails, memoranda, reports (including those with study data), presentations, meeting minutes, and other documents not originally intended to be publicly available. If documents were prepared for outside entities that were not employees or subcontractors, then we did not consider them to be internal company documents (e.g., we did not consider clinical trial protocols which are shared with institutional review boards and investigators to be internal company documents, nor did we include published research performed by company staff or contractors). We defined public health and healthcare in a broad sense to include studies of incidence, prevalence, etiology, prevention, diagnosis, harm, or prognosis, as well as any other studies concerning products or materials with health effects. Eligible studies could be qualitative (including descriptive and exploratory studies) or quantitative primary studies. While we recognize that systematic reviews may include additional information from internal company documents, we did not include them because this would have necessitated first, identifying all systematic reviews, and then second, checking each of them to see whether they used internal company documents.

### Search methods

#### Initial search efforts

Initially, we used the authors' combined file of articles meeting our eligibility criteria (n = 35 articles using pharmaceutical, tobacco and other industry documents) as a “reference set” against which we tested various search strategies. Four of the authors (NM, SSV, LR, and CNK) developed a search strategy using relevant Medical Subject Heading (MeSH) terms and title and abstract text words from the reference set. This initial search retrieved about 2 million citations in PubMed and, considering this too many citations to review, we started over.

#### Approach finally used to search electronically for eligible studies

Working with an informationist (LMR), the team revised the PubMed search strategy by identifying a more targeted combination of keywords and MeSH headings from the reference set and running separate searches. Examples of MeSH headings included industry[majr], disclosure[mesh], and access to information[mesh], and keywords included terms such as “industry documents” (see Appendix S1 for search strategy). PubMed was searched on December 21, 2010 and updated PubMed and Embase searches were run on April 7, 2011. These searches, taken together, we termed “the search finally used” and it yielded 9,305 citations after removal of duplicate records. We did not restrict the search finally used by language, year of publication, or type of publication.

We also report in the Results section an additional search we did after this one, in an attempt to find more articles that used internal pharmaceutical company documents. We based this additional search on articles found by the electronic search strategy finally used, described above.

### Screening, data extraction, and data analysis

To determine whether articles met inclusion criteria for our study, two authors independently screened the title and abstract of each citation and then independently examined the full text of each article considered unclear or possibly eligible as a result of screening. Differences in opinion were resolved through discussion.

Two authors independently extracted data from eligible English-language articles using a standardized online data extraction form. Articles not in English were assigned to a single data extractor with expertise in the language. We extracted information on the following items: language and date of publication, type of company (e.g., tobacco, pharmaceutical); source(s) of internal documents (e.g., litigation, U.S. Freedom of Information Act (FOIA) request); type of research question (e.g., about strategic behavior on the part of a company or industry, about effects of a therapeutic intervention); type of internal documents (e.g., research studies, internal memos); and funding source (e.g., government, non-profit, for-profit) for the study (see Appendix S2 for data abstraction form). Discrepancies in data extraction were resolved through discussion. One author extracted additional details about funding source(s) after the initial data abstraction was completed.

When an included article focused on research methods, one author classified the article into one of the following categories: 1) criticism of industry research (e.g., suggestion of misconduct or problems with dissemination of research), 2) exploration of company research methods that was not focused on criticism of the company, or 3) exploration of methods for accessing or analyzing internal company documents for the purposes of non-company research. A second author verified the classification with disagreements resolved through discussion.

One author collected additional details via email correspondence with authors of pharmaceutical research articles when the articles contained insufficient or unclear information about the location of internal company documents. These details were abstracted into a table, and a second author read the emails to verify the abstraction.

One author compared the results of our searches to known reference standards of research articles using internal company documents. For tobacco articles, we used the online Tobacco Documents Bibliography at the Tobacco Control Archives held by the University of California, San Francisco (UCSF) [26] as the reference standard. We compared articles classified in the Bibliography as “journal articles” and dated March 2011 or earlier against articles in our search results.

For research articles using internal company documents for other types of companies, we were not aware of a source we could use as a true reference standard. We were particularly interested in identifying articles using pharmaceutical company documents and therefore applied three methods to identify additional articles we might have missed through our electronic searches.

First, we used *Science Citation Index – Web of Science* to retrieve citations to our included pharmaceutical company articles, limiting citations to those published in March 2011 or earlier. Two authors independently screened the citation titles and abstracts for eligibility and independently examined the full text if eligibility was unclear or probable. All differences were resolved through discussion or consultation with a third author.

Second, one author visited the website of the Drug Industry Document Archive (DIDA) [27] and checked the Resources page (dida.library.ucsf.edu/resources.jsp) for potentially eligible journal articles published in March 2011 or before. A second author confirmed the eligibility classification.

Finally, we retrieved a few potentially eligible articles through *ad hoc* means (e.g., through authors of included articles using pharmaceutical documents), and two authors agreed upon final eligibility classification. Articles that we identified though these three methods, plus the pharmaceutical company studies we had already identified, were considered a “reference standard,” understanding that they were a more complete set of articles using internal pharmaceutical company documents and not a true reference standard.

We performed descriptive statistical analyses, including counts of the number of studies with different characteristics, and cross tabulations of the joint distribution of study characteristics.

## Results

**Figure 1:**
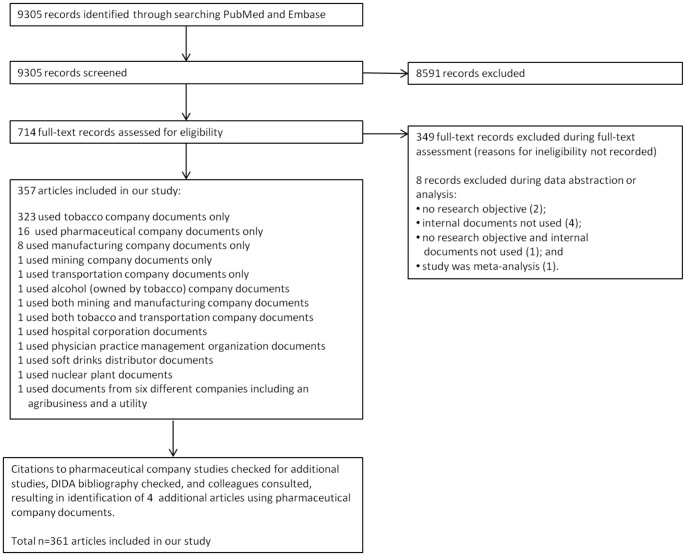
Flow chart of articles through the screening process.

Our search of PubMed and Embase retrieved 9,305 unique records. After screening, 357 articles were classified as eligible for the study. Our searches of other sources to identify additional articles using pharmaceutical documents identified two from *Science Citation Index – Web of Science*, one from DIDA, and one via *ad hoc* contact with colleagues. We therefore analyzed a total of 361 articles (see Figure 1 and Appendix S3). The great majority of articles were conducted using internal tobacco company documents (325/361; 90%). Others used documents from pharmaceutical companies (20/361; 6%), manufacturing companies (9/361; 2%), mining companies (2/361; <1%), transportation companies (2/361; <1%), alcohol companies (1/361; <1%), and other companies (5/361; 1%) (see Table 1). Three articles reported studies that used documents from more than one type of company, as described in table legends. Studies were published between 1982 and April 2011. All but six articles (355/361; 98%) were published in English: two were in Spanish and one each in French, German, Portuguese, and Swedish.

**Table 1:** Source of documents for articles using internal documents from different types of companies (n = 361 articles).

Source of internal documents	Tobacco	Pharmaceuticals	Manufacturing	Mining	Transportation	Alcohol	Other companies^1^	Total
	N (%)	N (%)	N (%)	N (%)	N (%)	N (%)	N (%)	N (%)^2^
Litigation^3^	324 (>99)	18 (90)	6 (67)	1 (50)	1 (50)	1 (100)	1 (20)	351 (97)
U.S. Freedom of Information Act (FOIA)	2 (1)	0 (0)	0 (0)	0 (0)	1 (50)	0 (0)	0 (0)	2 (1)
Company	0 (0)	2 (10)	2 (22)	0 (0)	0 (0)	0 (0)	4 (80)	8 (2)
Whistleblower	1 (<1)	0 (0)	0 (0)	0 (0)	0 (0)	0 (0)	0 (0)	1 (<1)
Unknown	0 (0)	0 (0)	1 (11)	1 (13)	0 (0)	0 (0)	0 (0)	1 (<1)
Other sources^4^	1 (<1)	0 (0)	0 (0)	0 (0)	1 (50)	0 (0)	0 (0)	2 (<1)
Total^5^	325 (100)	20 (100)	9 (100)	2 (100)	2 (100)	1 (100)	5 (100)	

1 The articles using documents from other companies were one article each using documents from a hospital, a physician practice management organization, a soft drinks distributor, and a nuclear plant, and one article using internal documents from six different companies including an agribusiness and a utility company.

2 The totals in this column equal the number of articles relying upon a particular source of documents, minus three instances of duplicate classification by type of company within category of document source. These instances were: one article with litigation source was classified as both tobacco and alcohol; one article with FOIA source was classified as both tobacco and transportation; and one article with unknown source was classified as both manufacturing and mining. The overall column total is not shown, as it is greater than the total number of included articles (n = 361) because several articles relied upon multiple sources for documents.

3 The litigation-related source of documents for one pharmaceutical article was a leak from legal proceedings.

4 The other sources of documents were: private archives of a company consultant (1 tobacco article) and records from a bankruptcy (1 transportation article).

5 The totals in this row equal the number of articles for each type of company, minus instances of duplicate sources of documents. Two tobacco articles relying upon FOIA for documents and one tobacco article relying upon other sources of company documents (the private archives of a company consultant) also relied upon documents from litigation, and one transportation article relied upon both litigation and FOIA. The totals for the tobacco and transportation article columns are therefore not equal to the sum of the classifications within the columns. The overall row total is not shown, as it is greater than the total number of included articles (n = 361) because three articles were classified with two types of companies.

Most included articles relied upon internal company documents obtained through litigation (350/361; 97%) (see Table 1). The single tobacco company article not using documents from litigation used documents provided by a whistleblower, and those documents were later also released through litigation. Litigation was also the primary source of internal company documents from the pharmaceutical industry (18/20; 90%). Cooperation with the company being studied was the next most common source of documents (9/361; 2%).

The research questions posed in included articles were primarily about company strategies to promote or position the company and its products (326/361; 90%) (see Table 2 and Appendix S2). Research questions about research methods were posed only by tobacco or pharmaceutical company articles; all pharmaceutical company articles in this category focused upon company misconduct (9/9; 100%), as did the majority of tobacco company articles (18/31; 58%).

**Table 2:** Research questions for articles using internal documents from different types of companies (n = 361 articles).

Types of questions	Tobacco	Pharmaceuticals	Manufacturing	Mining	Transportation	Alcohol	Other	Total questions
	N (%)	N (%)	N (%)	N (%)	N (%)	N (%)	N (%)	N (%)^1^
Company's strategic behavior (eg, marketing)	303 (93)	15 (75)	6 (67)	2 (100)	1 (50)	1 (100)	1 (20)	326 (90)
Company's other behavior (eg, safety)	7 (2)	1 (5)	3 (33)	0 (0)	0 (0)	0 (0)	1 (20)	12 (3)
Health effects of exposure or intervention	19 (5.9)	6 (30)	5 (56)	0 (0)	1 (50)	0 (0)	1 (20)	33 (9)
Therapeutic intervention	1 (<1)	7 (35)	0 (0)	0 (0)	1 (50)	0 (0)	0 (0)	9 (2)
Prevalence of intervention, exposure or outcome	2 (1)	0 (0)	2 (22)	0 (0)	0 (0)	0 (0)	2 (40)	6 (2)
Research methods	31 (10)	9 (45)	0 (0)	0 (0)	0 (0)	0 (0)	0 (0)	40 (11)
Total^2^	325 (100)	20 (100)	9 (100)	2 (100)	2 (100)	1 (100)	5 (100)	

1 The totals in this column equal the number of articles asking a particular type of question, minus instances of duplicate classification by type of company within category of type of question. These instances were: Strategic behavior questions were asked by articles classified as both tobacco and transportation, both mining and manufacturing, and both tobacco and alcohol; a therapeutic intervention question was asked by the article classified as both tobacco and transportation. The overall column total is not shown, as it is greater than the total number of included articles (n = 361) because several articles posed multiple types of questions.

2 The totals in this row equal the total number of articles for each type of company, minus instances where articles asked multiple types of questions, of which there are too many to list. The totals for the columns are therefore not equal to the sum of the classifications within the columns. The overall row total is not shown, as it is greater than the total number of included articles (N = 361) because three articles were classified with two types of companies.

It was often difficult to identify the exact type of internal company documents used in the articles. Our interest was not in the format of the document but rather in the type of document information that appeared to have been used in the article. We were interested in whether the document contained quantitative study data and if so whether the data was produced by the company itself or by another entity acting on behalf of the company. We also wished to capture whether the document was the result of routine company activities. We therefore classified the type of document information as belonging to one or more of four categories: 1) quantitative study data from internal company studies (e.g., analysis or re-analysis of quantitative data from studies conducted by the company), 2) quantitative data from non-company studies (e.g., quantitative data quoted from market research conducted on behalf of a company), 3) data from company records collected as part of routine company activities (e.g., employee records), and 4) ‘other’ types of data, information generally from miscellaneous documents such as memos or letters, or from unspecified types of documents. Most studies in the review (344/361; 95%) were classified as using ‘other’ types of company data (see Table 3), with only 20% overall using quantitative data. Use of quantitative data varied quite a bit across the types of companies examined, however, with almost half (9/20) of studies using pharmaceutical company documents accessing quantitative data.

**Table 3:** Types of internal company data/information in articles using internal documents from different types of companies (n = 361 articles).

Types of internal company data	Tobacco	Pharmaceuticals	Manufacturing	Mining	Transportation	Alcohol	Other	Total
	N (%)	N (%)	N (%)	N (%)	N (%)	N (%)	N (%)	N (%)^1^
Quantitative data from company study	61 (19)	9 (45)	3 (33)	1 (50)	0 (0)	0 (0)	0 (0)	73 (20)
Quantitative data from non-company study	38 (12)	0 (0)	1 (11)	0 (0)	0 (0)	0 (0)	0 (20)	39 (11)
Data from company day-to-day records	3 (1)	2 (10)	3 (33)	0 (0)	1 (50)	0 (0)	5 (100)	14 (4)
Other types of company data/information or type is unclear	321 (99)	14 (70)	6 (67)	2 (100)	1 (50)	1 (100)	4 (80)	344 (95)
Total^2^	325 (100)	20 (100)	9 (100)	2 (100)	2 (100)	1 (100)	5 (100)	

1 The totals in this column equal the number of articles using a particular type of data, minus instances of duplicate classification by type of company within category of type of data. These instances were: Other types of data were used by articles classified as both tobacco and transportation, both mining and manufacturing, and both tobacco and alcohol, and quantitative data from internal company studies were used by the article classified as both mining and manufacturing. The overall column total is not shown, as it is greater than the total number of included articles (n = 361) because several articles used multiple types of internal documents.

2 The totals in this row equal the total number of articles for each type of company, minus instances where articles used multiple types of data, of which there are too many to list. The totals for the columns are therefore not equal to the sum of the classifications within the columns. The overall row total is not shown, as it is greater than the total number of included articles (N = 361) because three articles were classified with two types of companies.

Articles describing studies using tobacco company documents consistently referred to physical or online tobacco company document repositories as the location of the documents used in the study. Articles describing studies using pharmaceutical company documents did not have a consistent source of documents, and we investigated the current location of those documents. Of the 20 articles using internal pharmaceutical company documents, two used documents made available directly to the researchers by the company, and the remainder used documents released as a result of litigation (n = 18, including one case in which documents were leaked from litigation) (see Table 4). There is substantial overlap in the litigation, authors, and/or documents used in these articles, although this is sometimes because of multiple instances of related litigation, not because the same documents were used (e.g., Vedula 2009 examined documents from multiple Neurontin litigations; and separate documents from one of these litigations were also examined in Steinman 2006, Steinman 2007, and Landefeld 2009). The internal pharmaceutical company documents released as a result of litigation are not necessarily publicly available. Documents used in articles can be found in DIDA (9 articles); court records only (2, including 1 article where actual documents are not accessible although the report on the documents is accessible); and court records plus website (4 articles with active website links and 3 articles citing non-working links).

**Table 4:** Sources and location of pharmaceutical internal company documents.

Company and product name^1^	Source(s) of documents according to article/correspondence with author	Location(s) of documents as of May 10, 2013	Article(s)
**Documents from litigation sources (n = 18), ordered by company name and document group** 1			
Bayer: cerivastatin (Baycol).	Documents from litigation. Documents part of the public record through Hollis N. Halton v. Bayer, Nueces County Clerk, Tx.	Documents part of court records. No online link to documents.	Psaty et al. 2004 [35]
Eli Lilly: olanzapine (Zyprexa).	Documents from litigation, available at http://www.furiousseasons.com/zyprexadocs.html.	Documents part of court records. Online link to documents not active as of 5/10/13.	Applbaum 2009 [43]
Eli Lilly: olanzapine (Zyprexa).	Documents leaked from litigation.	Author says the documents are available at http://www.furiousseasons.com/zyprexadocs.html. Documents part of court records. Online link to documents not active as of 5/10/13.	Spielmans 2009 [44]
Eli Lilly: olanzapine (Zyprexa).	Documents from litigation (two lawsuits). Unpublished analyses by sponsor of premarket safety data.	Documents currently available through http://zyprexakills.ath.cx/ and www.zyprexalitigationdocuments.com (or direct communication with the lawyers).	Woods et al. 2011 [45]
Glaxo Smith Kline (GSK): paroxetine (Paxil)	Documents from litigation. The expert report was based on 3-day examination of files at company headquarters by Dr. Breggin.	Actual documents used are not publicly accessible, only psychiatric expert report. Report available at the court (Moffett v. GSK, United States District Court for the Southern District of Mississippi) and at http://breggin.com/index2.php?option=com_docman&task=doc_view&gid=20&Itemid=3	Breggin 2006 [46]
Glaxo Smith Kline (GSK): paroxetine (Paxil)	Documents from litigation. Authors had access to confidential documents as a consequence of their roles in litigation. Some documents in the case have been released into the public domain.	Documents part of court records for *The People of the State of New York vs. SmithKline Beecham Corp*. (Case No. 04-CV-5304 MGC) and *Beverly Smith vs. SmithKline Beecham Corp*. (Case No. 04 CC 00590). All documents referred to in the paper are available through www.healthyskepticism.org/documents/PaxilStudy329.php.	Jureidini et al. 2008 [37], Jureidini and McHenry 2011 [47], and McHenry and Jureidini 2008 [38]
Merck: rofecoxib (Vioxx)	Documents from litigation: *Humeston, et al. vs Merck & Company, Inc*. Case #619 Superior Court of New Jersey, Atlantic County, New Jersey (February 6, 2007). One of the authors, an expert for the plaintiff's attorneys in litigation against Merck, reviewed internal company documents and re-analyzed datasets submitted by the company to the FDA. Article states that “all legal documents cited in this article were said to be available at http://www.biostat.washington.edu/research/Rofecoxib.”	Documents part of court records. Online link to documents not active as of 5/10/13.	Psaty and Kronmal 2008 [39]
Merck: rofecoxib (Vioxx)	Documents available through litigation. Authors had access to internal Merck documents created in 1998–2006, and obtained through discovery in legal proceedings, *Cona v. Merck* and *McDarby v. Merck*.	All legal documents and the dataset used in the first three articles are available in the Drug Industry Document Archive (DIDA) at http://dida.library.ucsf.edu. The documents, but not the dataset, for Ross et al. 2010 are available in DIDA.	Hill et al. 2008 [23], Krumholz et al. 2007 [48], Ross et al. 2008 [40], and Ross et al. 2010 [49]
Pfizer & Parke-Davis, Division of Warner-Lambert: gabapentin (Neurontin).	Documents from litigation. Authors obtained access to the data because they served as unpaid expert witnesses for the plaintiff in the whistleblower litigation *United States of America ex. rel David Franklin vs. Pfizer, Inc., and Parke-Davis, Division of Warner-Lambert Company*.	Documents available in DIDA at http://dida.library.ucsf.edu.	Steinman et al. 2006^2^ [36], Steinman et al. 2007^2^ [50], and Landefeld and Steinman 2009[51].
Pfizer & Parke-Davis, Division of Warner-Lambert: gabapentin (Neurontin).	Documents from litigation. Authors obtained access to the data because two of them served as consultants for the plaintiff in *Re: Neurontin Marketing, Sales Practices, and Products Liability Litigation, United States District Court, District of Massachusetts*.	All of these documents are available in DIDA at http://dida.libriary.ucsf.edu or are available in the Clerk's office for the United States District Court in Boston, Massachusetts and may be accessed at https://ecf.mad.uscourts.gov/doc1/09502786849	Vedula et al. 2009^2^ [4]
Wyeth: conjugated equine estrogens and medroxyprogesterone acetate (Prempro)	Documents from litigation. Expert report by Dr. Fugh-Berman based on documents from Wyeth interactions with DesignWrite (medical education and communications company). Documents used in expert MDL Docket no 4:03CV1507 WRW, and used again in *Scroggin v. Wyeth (in re: Prempro Products Liability Litigation), 586 F.3d 547, (8th Cir. 2009), United States Court of Appeals*.	Prempro Products Liability Litigation now available at http://www.plosmedicine.org/static/ghostwriting.action or in DIDA at http://dida.libriary.ucsf.edu	Fugh-Berman 2010 [52]
**Documents provided by company to researchers (n = 2)**			
**Company and product name** 1	**Source(s) of documents according to article/correspondence with author**	**Location(s) of documents as of May 10, 2013**	**Article(s)**
Merck: MDMA (“ecstasy”).	Merck Archives. Internal company documents recording the history of drug development.	Internal documents not publicly available.	Bernschneider-Reif et al. 2006 [53]
Merck, Sharpe and Dohme: chlorothiazide (Diuril).	Merck Archives. Internal company documents recording the history of development and promotion of chlorothiazide.	Internal documents not publicly available.	Greene 2005 [54]

1 Documents are grouped in rows where the articles are linked by a common set of authors working with the same set of documents.

2 Vedula and colleagues (Vedula et al 2009) included in their analysis internal company documents from a 2004 litigation that were also used by other authors in two articles (Steinman et al 2007 and Landefeld and Steinman 2009), and in addition analyzed documents from a 2008 litigation that were not used in other articles.

Explicit information about study funding was found in 290/361 (80%) articles, of which a small number (10/290; 3%) specified that the study had not been funded and 280 listed funding. Among the 280 articles describing funding received specifically for the study, the most common source of funding was the U.S. government, followed by non-profit organizations (see Table 5). The majority of studies reporting U.S. government funding reported that some or all of the government funding was from the National Cancer Institute (NCI) at the US National Institutes of Health (225/248; 91%), and aside from one study using documents from the nuclear industry, the NCI-funded studies all examined documents from the tobacco industry. Overall, 224/361 (62%) studies in this review used tobacco documents from litigation and were funded by the NCI. Seven articles using tobacco company documents, and one article using pharmaceutical company documents, mentioned that the authors or their institutions received funding to develop archives of documents related to their investigation.

**Table 5:** Types of funding reported by research articles using internal documents from different types of companies (n = 361articles).

Type of funding	Tobacco	Pharmaceuticals	Manufacturing	Mining	Transportation	Alcohol	Other	Total^1^
	N (%)	N (%)	N (%)	N (%)	N (%)	N (%)	N (%)	N (%)
No information about how research using documents was funded	54 (17)	9 (43)	4 (44)	2 (100)	1 (50)	0 (0)	2 (40)	71 (20)
Statement that research using documents was not funded	2 (1)	8 (67)	0 (0)	0 (0)	0 (0)	0 (0)	0 (0)	10 (3)
Funding from government, US	242 (74)	3 (15)	1 (11)	0 (0)	0 (0)	0 (0)	2 (40)	248 (69)
Funding from government, not US	36 (11)	0 (0)	0 (0)	0 (0)	0 (0)	0 (0)	0 (0)	36 (10)
Funding from non-profit organization	80 (25)	3 (17)	2 (22)	0 (0)	1 (50)	1 (100.0)	1 (20)	86 (24)
Funding from company being studied	0 (0)	0 (0)	1 (11)	0 (0)	0 (0)	0 (0)	1 (20)	2 (<1)
Funding from other sources^2^	6 (2)	0 (0)	1 (11)	0 (0)	0 (0)	0 (0)	0 (0)	7 (2)
Total^3^	325 (100)	20 (100)	9 (100)	2 (100)	2 (100)	1 (100)	5 (100)	

1 The totals in this column equal the number of articles reporting a particular type of funding, minus instances of duplicate classification by type of company within funding category. These instances were: There was no information on funding for the article classified as both manufacturing and mining, and non-profit, non-governmental funding was used by the articles classified as both tobacco and transportation and both tobacco and alcohol. The overall column total is greater than the total number of included articles (N = 361) because some articles reported multiple types of funding.

2 Other funding sources include Blue Cross Blue Shield (4 tobacco articles), the World Health Organization (2 tobacco articles), and funding from a law firm (1 manufacturing article).

3 The totals in this row equal the total number of articles reporting funding for each type of company, minus instances where articles reported multiple types of funding, of which there are too many to list. The totals for the columns are therefore not equal to the sum of the classifications within the columns. The overall row total is greater than the total number of included articles (N = 361) because three articles were classified with two types of companies.

The source of our reference standard for tobacco studies, the Tobacco Documents Bibliography, contained 579 journal articles published in March 2011 or earlier. Our searches identified 337/579 (58%) of these records, of which 307/337 (91%) were deemed eligible and included in our study. Of the 242 remaining journal articles contained in the reference standard, 173 were indexed in PubMed or Embase and were not captured by our searches. On the other hand, 18/325 (6%) of the tobacco company records in our study were not included in the Tobacco Documents Bibliography.

Given the low number of citations reporting research using internal pharmaceutical company documents that were captured by the search we finally used, an informationist designed an additional strategy tailored to be more sensitive and to identify research using internal pharmaceutical company documents, and a second informationist reviewed the strategy. Eighteen (two later determined to be not eligible) pharmaceutical company research articles (15 PubMed records and 3 Embase) retrieved by our original search formed the basis for this “drug industry” search strategy. One author reviewed the reference lists of the 18 articles and selected references on the topic of internal pharmaceutical company documents (n = 53), and a colleague provided a list of additional related articles (n = 8). Keywords and MeSH terms from the 18 originally included articles, the 53 selected references, and the 8 related articles were combined into a more targeted and potentially more sensitive search strategy, which was run in PubMed. This search strategy captured 17/18 of the PubMed citations to the pharmaceutical company articles finally included in our study. To achieve this level of sensitivity, however, the new, more sensitive PubMed search identified 26,399 “hits”, of which 25,605 had not been identified by the previous search, and we decided that this was an unmanageable number for continuing to search for eligible articles.

## Discussion

Internal company documents serve as a valuable source of information about industry activities for those who wish to know about the impact of those activities upon the health of the public. Internal documents from pharmaceutical companies include not only information on marketing and policy activities but also contain quantitative and other data related to clinical trials carried out on company products. Data from all trials are critical for a complete and accurate assessment of interventions within systematic reviews [15], [19], [28]. In response to this imperative, systematic reviewers and other healthcare stakeholders have been working hard towards making trial data held by companies publically available [29], [30] so that patients, providers, and policy makers can have a full picture of all that is known. Because internal company documents are not located in a single place, and they are not published or indexed in a bibliographic database, they are difficult to identify and locate. We elected to identify research articles using internal company documents across all industries as a starting point, knowing that we would likely leave many documents that are available for research unidentified.

What we learned, first, is that thousands of internal tobacco company documents, mainly released through litigation, are located in repositories around the world [31], including searchable online repositories (e.g., the Legacy Tobacco Documents Library at legacy.library.ucsf.edu and Tobacco Documents Online at tobaccodocuments.org) [32]. Ninety percent (325/361) of the research articles meeting our criteria used internal tobacco company documents and all but one of these used documents made available through litigation. The reference standard we used for articles employing internal tobacco documents indicated that our searches failed to find hundreds of additional, potentially eligible, studies of tobacco documents and that our searches also identified tobacco articles not included in the Tobacco Documents Library.

The second thing we learned was that identification of non-tobacco studies using internal company documents was harder than we had anticipated. Only 36/361 articles that we identified used non-tobacco sources, and more than half of these (20/36) were concerned with pharmaceutical company documents. We made every effort to ensure a thorough search of PubMed and Embase databases to retrieve all relevant documents, and to be practical we designed a search strategy that elected precision over sensitivity. It is possible that there are additional relevant articles from other non-tobacco industries (e.g., the chemical industry, the food and agricultural industry) that our search failed to retrieve. We need to identify better search terms for retrieving articles that use internal company documents, and consider consistent indexing of such articles. New machine learning approaches to searching databases may be a way to improve the retrieval of difficult-to-find articles as well. We also found that in contrast to tobacco company documents, which are contained in well-indexed repositories developed to facilitate public access to information, the pharmaceutical company documents are available in a range of sites, not all of which are well-known or accessible to the public. In addition, bibliographies of articles using internal pharmaceutical company documents, similar to the Tobacco Documents Bibliography, would greatly ease the identification of research using internal pharmaceutical company data.

These findings point to the importance of having one or more indexed and searchable repositories in place to assure comprehensive identification of internal company documents. Litigation has been an important source of internal company documents for research, and some documents from pharmaceutical company litigation have now been placed in DIDA; indeed, DIDA was started with funds from litigation. Nevertheless, the majority of pharmaceutical company documents in the studies we found were made available through websites (some no longer accessible), were obtained through collaboration with the company, or are court documents that one must know exist to be able to find. Comprehensive well-indexed and searchable repositories of internal company documents from pharmaceutical and other industries, similar to the repositories that exist for documents from the tobacco industry, are critical for the development of a program of research using other types of internal documents, including restorative authorship [21]. One or more repositories of internal documents from pharmaceutical companies, including trial data, will become critically important should the European Medicines Agency adopt the policy of requiring pharmaceutical company release of clinical trial data [33]. Until such time as well-populated, well-indexed, and well-publicized repositories are developed, the best advice for those seeking to find and use internal documents and related unpublished documents (e.g., regulatory documents) from pharmaceutical companies is to follow the activities outlined by Chan in 2012 [34].

The third thing we learned is that funding for research using internal company documents is uneven. Where there has been funding available, notably for the tobacco-related research, many important research projects have been conducted. Three-quarters of the tobacco research was funded by the U.S. government, primarily the NCI. Indeed, the NCI established a program of research and actively solicited researchers to develop projects using internal tobacco documents (e.g., http://grants.nih.gov/grants/guide/pa-files/PAR-01-063.html). From 2000 to 2007, the NCI provided a total of approximately $23 million to fund tobacco industry document research studies [personal written communication, Tobacco Control Research Branch Behavioral Research Program, Division of Cancer Control and Population Sciences, NCI, July 12 2013].

In contrast, research studies making use of internal pharmaceutical company documents have typically not been federally funded. Most articles we identified (13/20) reported no funding or had no explicit information about funding for the research, and only 3/20 reported US state or federal government funding. If, to date, only a handful of research studies have used internal pharmaceutical company documents, then it may be because of lack of available funding. Given the importance of research using internal tobacco documents to our current knowledge and views about tobacco and its health effects, a similar investment in other areas, including the pharmaceutical area, could also yield potentially important findings.

We do not know whether our initial search would have found more, fewer, or the same number of studies using pharmaceutical documents in our reference standard, if we had followed through and screened the over 2 million citations retrieved. It is possible that the number of research articles using pharmaceutical company documents is actually small and that we found most of them. We know that of the articles we identified, there was considerable overlap in documents, authors, and drugs examined. If we have identified most of the relevant articles, it highlights all there is to be gained by making all publicly available source documents (especially clinical study reports and datasets) accessible in one or a few locations, assuming this will prompt new research [22].

The studies of internal pharmaceutical company documents we identified, and others, have provided important signals for evidence-based medicine, indicating that the published literature, generally, is not always reliable and that much of what is known remains unpublished [4], [35], [36], [37], [38], [39], [40]. Society expects scientific studies to be conducted and disseminated following generally accepted tenets of scientific integrity and to adhere to a code of research ethics. We found that 9/20 studies using internal pharmaceutical company documents examined research methods used by the company, and all of these studies (9/9) were critical of the scientific and ethical integrity of the companies' research. Most of the research articles we identified examined strategies used by pharmaceutical companies to achieve commercial goals, which runs counter to scientific research goals.

While our particular interest in this project was pharmaceutical company documents, other company documents released through litigation or other means and potentially useful for health-related research and for setting governmental standards (e.g., regarding environmental hazards) should also be made centrally available to researchers. These collections of corporate documents should ideally be linked or merged, as companies often collaborate across industries (e.g., large corporations control both tobacco and alcohol companies) to promote their interests, often at the expense of public health [41]. Studies of these activities would be facilitated if searching could be done across several industries. Current methods for identifying internal company documents from litigation and other sources, with or without study data and CSRs, include word of mouth, unstructured searches of the internet, and, in the US, searching the Public Access to Court Electronic Records (PACER) system (www.pacer.gov). All of these methods are of uncertain reliability, sensitivity, and precision. Our findings are similar to those from almost 25 years ago that retrospective searching for unpublished trials was not useful, and that a comprehensive register of all initiated trials was needed [42].

Our study is limited by our focus on relevant research indexed in PubMed or Embase, and by a search date that is now more than three years in the past. However, while our study is limited by the possibility that we overlooked relevant research using internal company documents, including documents used in systematic reviews, we are able to conclude that our findings highlight the great need for well-indexed and curated repositories so that researchers can have ready access to internal company documents. The existing DIDA repository is a good start but additional funds are required to make it maximally useful to researchers. Each document in the repository needs to have consistent indexing information (metadata) such as title, author, date, bates number, and document type. This would either need to be provided (e.g., by the plaintiffs' attorneys) or a vendor would have to be hired to create it. In addition, funding is needed for DIDA's ongoing curation to support, for example, information science and programming staff. Linking repositories and bibliographies (e.g., unpublished data in systematic reviews) should be explored, as well as linking these sources and registers of studies (e.g., ClinicalTrials.gov and the Cochrane Register of Studies). The research articles we identified relating to tobacco industry documents are a testament to how information in internal company documents can contribute to improving the community's understanding of enhancing transparency in communicating research findings.

## Supporting Information

Appendix S1 **Search strategy.**
(DOCX)

Appendix S2 **Data abstraction form.**
(PDF)

Appendix S3 **List of all included articles.**
(DOCX)
