# Supplementary material for: Who Has Used Internal Company Documents for Biomedical and Public Health Research and Where Did They Find Them?
Source: PLoS One. 2014 May 6;9(5):e94709. doi: 10.1371/journal.pone.0094709 (PMC4011692; doi:10.1371/journal.pone.0094709)
Supplement: Appendix S1 — Search strategy. (DOCX) [file pone.0094709.s001.docx]

Appendix 1. Search strategy to identify articles using internal company documents for health research

PubMed:  
(Industry[majr] AND (legislation and jurisprudence [Subheading] OR liability, legal[mesh]) AND (disclosure[mesh] OR publication bias[mesh] OR information dissemination[mesh] OR access to information[mesh])) OR ("internal document"[tiab] OR "internal documents"[tiab] OR (internal[tiab] AND company[tiab] AND analyses[tiab]) OR "industry documents"[tiab] OR "court documents"[tiab] OR "industry research"[tiab] OR litigation[tiab] OR (internal[tiab] AND corporate[tiab] AND document[tiab]) OR "internal research"[tiab] OR "internal company documents"[tiab] OR (internal[tiab] AND company[tiab] AND document[tiab]) OR (internal[tiab] AND research[tiab] AND documents[tiab]) OR "destroyed documents"[tiab] OR "legal proceeding"[tiab] OR "legal proceedings"[tiab] OR "legal document"[tiab] OR "legal documents"[tiab] OR(documents[tiab] AND library[tiab]) OR "document destruction"[tiab] OR "documentary evidence"[tiab] OR "company documents"[tiab] OR (company[tiab] AND document[tiab]) OR "private papers"[tiab] OR (discovery[tiab] AND documents[tiab]) OR (internal[tiab] AND scientific[tiab] AND documents[tiab]) OR (internal[tiab] AND scientific[tiab] AND document[tiab]) OR "confidential report"[tiab] OR "confidential reports"[tiab] OR "internal memoranda"[tiab] OR (internal[tiab] AND memorandum[tiab]) OR (internal[tiab] AND scientific[tiab] AND research[tiab]) OR "industry-linked"[tiab])
******************************************
Embase:  
('industry'/exp/mj) AND ('law'/exp OR 'law suit'/exp OR 'jurisprudence'/exp OR 'legal liability'/exp) AND ('interpersonal communication'/exp OR 'publishing'/exp OR 'information dissemination'/exp OR 'access to information'/exp) OR ("internal document":ti:ab  OR "internal documents":ti:ab OR (internal:ti:ab  AND company:ti:ab  AND analyses:ti:ab) OR "industry documents":ti:ab  OR "court documents":ti:ab  OR "industry research":ti:ab  OR litigation:ti:ab  OR (internal:ti:ab  AND corporate:ti:ab  AND document:ti:ab) OR "internal research":ti:ab  OR "internal company documents":ti:ab  OR (internal:ti:ab  AND company:ti:ab  AND document:ti:ab) OR (internal:ti:ab  AND research:ti:ab  AND documents:ti:ab) OR "destroyed documents":ti:ab  OR "legal proceeding":ti:ab  OR "legal proceedings":ti:ab  OR "legal document":ti:ab  OR "legal documents":ti:ab  OR (documents:ti:ab  AND library:ti:ab) OR "document destruction":ti:ab  OR "documentary evidence":ti:ab  OR "company documents":ti:ab  OR (company:ti:ab  AND document:ti:ab) OR "private papers":ti:ab  OR (discovery:ti:ab  AND documents:ti:ab) OR (internal:ti:ab  AND scientific:ti:ab  AND documents:ti:ab) OR (internal:ti:ab  AND scientific:ti:ab  AND document:ti:ab) OR "confidential report":ti:ab  OR "confidential reports":ti:ab  OR "internal memoranda":ti:ab  OR (internal:ti:ab  AND memorandum:ti:ab) OR (internal:ti:ab  AND scientific:ti:ab  AND research:ti:ab) OR "industry-linked":ti:ab)
*********************
