# Supplementary material for: Who Has Used Internal Company Documents for Biomedical and Public Health Research and Where Did They Find Them?
Source: PLoS One. 2014 May 6;9(5):e94709. doi: 10.1371/journal.pone.0094709 (PMC4011692; doi:10.1371/journal.pone.0094709)
Supplement: Appendix S2 — Data abstraction form. (PDF) [file pone.0094709.s002.pdf]

Data Extraction Form for Internal Company Data

Identifying information

This is the data abstraction form for the systematic review of reports using data from internal company documents.

You can move back and forth in the data abstraction form to change your answers. However, once you close your browser, click the "Exit this survey" link, or click the "Done" button, you cannot go back into the form to edit your answers. Should you wish to change your data abstraction, you will have to re-enter all the data for that report in a new session.

This page collects identifying information about the data abstractor and the article being abstracted. An asterisk indicates that an answer is required.

* 1. Name of data abstractor

- ◯ Chris
- ◯ Claire
- ◯ Karsten
- ◯ Kay
- ◯ Lainie
- ◯ Lori
- ◯ Nancy
- ◯ Stephan
- ◯ Swaroop

Other (please specify)

[ ]

* 2. Article accession ID

[ ]

* 3. Last name of first author

[ ]

4. Year of publication (please leave blank if unable to tell)

[ ]

Data Extraction Form for Internal Company Data

*5. What is the language of the main text of the report?

- English
- French
- German
- Spanish
- Other (please specify)

Data Extraction Form for Internal Company Data

Eligibility check

This page contains questions to confirm that the report is eligible for the systematic review.

***6. Is the topic of this report related to public health or health care? Examples include studies of incidence, prevalence, prognosis, intervention effectiveness or harm, etiology, and screening or diagnosis. Examples also include studies of business practices or regulations related to products that have or may have health effects.**

- ◯ Yes
- ◯ No

***7. Does the report appear to address a research question or have a research objective?**

- ◯ Yes
- ◯ No

***8. Were internal corporate documents used to provide data (information) examined in the study? (Internal corporate documents are company documents, such as emails, memoranda, reports, presentations, meeting minutes, etc., that were not intended to be publicly available.)**

- ◯ Yes
- ◯ No

***9. If all of the questions on this page are answered 'Yes', the article is eligible for the systematic review. If any of the questions on this page are answered 'No', the article is ineligible for the systematic review.**

Is the article eligible or ineligible?

- ◯ Eligible
- ◯ Ineligible

Data Extraction Form for Internal Company Data

Type of industry

This question asks you to specify the industry that was the source of the documents. The question should be answered based upon the information contained in the report.

10. What industry did the internal company documents come from? (You may select more than one answer, if necessary)

- ☐ Pharmaceutical
- ☐ Tobacco
- ☐ Alcohol
- ☐ Insurance
- ☐ Mining
- ☐ Manufacturing (eg, asbestos)
- ☐ Transportation
- ☐ Not described/Can't tell
- ☐ Other (please specify)

Data Extraction Form for Internal Company Data

How the internal company documents/data were released

This page has questions about how the internal company documents/data were released and became available to the researchers. Documents may have become available to the researchers in multiple ways. The question should be answered based upon the information contained in the report.

11. Based on the report, how does it appear that the internal company documents were made available to the researchers? (You may select more than one option, if necessary)

- [ ] Litigation or a Legal Settlement
- [ ] Freedom of Information Act (FOIA) request
- [ ] The Company (e.g., research in collaboration with the company)
- [ ] An Unauthorized Company Source (e.g., whistleblower)
- [ ] Not described/Can't tell
- [ ] Other (please specify)

Data Extraction Form for Internal Company Data

Type of research question

This page has questions about the type of research question contained in the report. A report may have multiple types of research questions. Please answer based upon the information contained in the report.

Each question has a 'Not Sure' option. This answer should be used when you think the answer may be 'Yes' but you do not see enough information in the report to support a 'Yes' answer. Each 'Not Sure' answer will be followed up individually, so please do not use it casually.

12. Was the research question about the strategic behavior of a corporation? Some examples of strategic behavior include: advertising, marketing or promotion, responses to research/health concerns, resistance to new or proposed laws or regulations, and design or manipulation of company products to promote the use of those products.

- ○ Yes
- ○ No
- ○ Not sure (please specify)

13. Was the research question about corporation behavior that is not related to strategy, promotion, or market positioning? An example of this behavior might be internal company implementation of safety-related industry regulations.

- ○ Yes
- ○ No
- ○ Not sure (please specify)

14. Was the research question about the health effects of corporation products or behaviors (e.g., health effects of a pharmaceutical product, health effects of modifications to cigarette design, health effects of exposures to industrial chemicals)?

- ○ Yes
- ○ No
- ○ Not sure (please specify)

15. Was the research question about the effects of a therapeutic intervention? A therapeutic intervention is an intervention focused on treatment or prevention. Examples of effects of a therapeutic intervention include benefits or harms of a drug or surgical device.

- ◯ Yes
- ◯ No
- ◯ Not sure (please specify)

16. Was the research question about the prevalence of an intervention, an exposure, or a disease? Examples of prevalence include patterns of use (e.g., of a medical drug or of tobacco) or patterns of disease (e.g., of lung cancer) in a population. Do not include patterns of intervention use or disease outcomes within an intervention study (e.g. a drug trial) in answering this question.

- ◯ Yes
- ◯ No
- ◯ Not sure (please specify)

17. Was the research question about research methods? Examples of research methods include how to obtain or analyze internal company data. Examples may also include examination of the conduct of research by companies or their associates (e.g., previously unknown research findings, use of ghost authorship).

- ◯ Yes
- ◯ No
- ◯ Not sure (please specify)

Data Extraction Form for Internal Company Data

Type of internal company data

This page has questions about the type of internal company data that was used to answer the research question. A report may use multiple types of internal company data. Please answer based upon the information contained in the report.

Each question has a 'Not Sure' option. This answer should be used when you think the answer may be 'Yes' but you do not see enough information in the report to support a 'Yes' answer. Each 'Not Sure' answer will be followed up individually, so please do not use it casually.

18. Did the report appear to use numerical data from studies conducted by the company itself (e.g., clinical trials or marketing research carried out by the company)? An example of this use is quoting or reanalyzing numerical data from a study.

- Yes
- No
- Not sure (please specify)

19. Did the report appear to use numerical company data from studies that were not conducted by the company itself (e.g., data from external studies funded by the company, or external research data that was reanalyzed by the company)? An example of this use is quoting or reanalyzing numerical data from a study.

- Yes
- No
- Not sure (please specify)

20. Did the report appear to use data from company records about day-to-day operations (e.g., personnel files, payroll files)? An example of this use of data might be be using internal records to classify employees into exposure categories for a workplace chemical.

- Yes
- No
- Not sure (please specify)

21. Did the report appear to use information from internal documents or internal reports, other than the types of data described in the previous questions? Examples of these other types of information include internal memos, company archives, and any unspecified internal documents. This type of "other" information or data also includes internal documents that refer to or are about studies, but are not actual study data.

- ◯ Yes
- ◯ No
- ◯ Not sure (please specify)

Data Extraction Form for Internal Company Data

Funding of the research project/report

The remaining questions are about the type of funding for the research, and should be answered based upon information contained in the report.

22. Did you find any information in the research report about funding?

- ◯ Yes
- ◯ No

Data Extraction Form for Internal Company Data

Statement of no funding

23. Did the authors explicitly state that they were not funded to carry out this research?

- ◯ Yes
- ◯ No

Data Extraction Form for Internal Company Data

Government funding of the research project/report

24. Did the authors state that they were funded to carry out this research by government (including federal, state, county, or municipal government) funding?

- ◯ Yes
- ◯ No

Non-profit funding of the research project/report

25. Was the government funding from the National Cancer Institute (NCI)?

- ◯ Yes
- ◯ No

26. Did the authors state that they were funded to carry out this research by funding that appeared to come from a non-profit source (e.g., a university, a non-governmental organization, a think tank, a foundation)?

- ◯ Yes
- ◯ No

Specific non-profit funding of the research project/report

27. Based on the report, where did the non-profit funding appear to come from? (You may select more than one source, if appropriate)

- [ ] University
- [ ] Foundation
- [ ] Think tank
- [ ] Doesn't specify/Don't know
- [ ] Other source (please specify)

Data Extraction Form for Internal Company Data

For-profit funding of the research project/report

28. Did the authors state that they were funded to carry out this research by funding that appeared to come from a for-profit source (e.g., a Corporation)?

- ◯ Yes
- ◯ No

Specific for-profit or other funding of the research project/report

29. Based on the report, where did the for-profit funding appear to come from? (You may select more than one source, if appropriate)

- [ ] Company/Corporation
- [ ] Trade Organization
- [ ] Doesn't specify/Don't know
- [ ] Other (please specify)

30. Based on the report, does it appear that the authors were supported by other funding (i.e., instead of or in addition to government, not-for-profit, or for-profit funding for the research)?

- ○ No
- ○ Yes (please specify)

Data Extraction Form for Internal Company Data

Comments

31. If you have any comments about the data report or the data abstraction please add them in the box below.
