# Supplementary material for: Who Has Used Internal Company Documents for Biomedical and Public Health Research and Where Did They Find Them?
Source: PLoS One. 2014 May 6;9(5):e94709. doi: 10.1371/journal.pone.0094709 (PMC4011692; doi:10.1371/journal.pone.0094709)
Supplement: Appendix S3 — List of all included articles. (DOCX) [file pone.0094709.s003.docx]

Appendix 3. Citations to all included articles (total 361 articles)

I. Articles using tobacco company documents (n=325)*

*Bond 2010 used documents from both tobacco and alcohol companies and Pion 2004 used documents from both tobacco and transportation companies.

1. Acevedo-Garcia D, Barbeau E, Bishop JA, Pan J, Emmons KM. Undoing an epidemiological paradox: the tobacco industry's targeting of US Immigrants. *Am J Public Health* 2004;94(12):2188-93.

2. Alechnowicz K, Chapman S. The Philippine tobacco industry: "the strongest tobacco lobby in Asia". *Tob Control* 2004;13 Suppl 2:ii71-8.

3. Alpert HR, O'Connor RJ, Spalletta R, Connolly GN. Recent Advances in Cigarette Ignition Propensity Research and Development. *Fire Technol* 2010;46(2):275-89.

4. Andersen BS, Begay ME, Lawson CB. Breaking the alliance: Defeating the tobacco industry's allies and enacting youth access restrictions in Massachusetts. *Am J Public Health* 2003;93(11):1922-8.

5. Anderson SJ, Glantz SA, Ling PM. Emotions for sale: cigarette advertising and women's psychosocial needs. *Tob Control* 2005;14(2):127-35.

6. Anderson SJ, Dewhirst T, Ling PM. Every document and picture tells a story: using internal corporate document reviews, semiotics, and content analysis to assess tobacco advertising. *Tob Control* 2006;15(3):254-61.

7. Anderson SJ, Pollay RW, Ling PM. Taking ad-Vantage of lax advertising regulation in the USA and Canada: reassuring and distracting health-concerned smokers. *Soc Sci Med* 2006;63(8):1973-85.

8. Anderson SJ, Ling PM. "And they told two friends...and so on": RJ Reynolds' viral marketing of Eclipse and its potential to mislead the public. *Tob Control* 2008;17(4):222-9.

9. Apollonio DE, Malone RE. Marketing to the marginalised: tobacco industry targeting of the homeless and mentally ill. *Tob Control* 2005;14(6):409-15.

10. Apollonio DE, Bero LA. The creation of industry front groups: the tobacco industry and "get government off our back". *Am J Public Health* 2007;97(3):419-27.

11. Apollonio DE, Malone RE. Turning negative into positive: public health mass media campaigns and negative advertising. *Health Educ Res* 2009;24(3):483-95.

12. Apollonio DE, Malone RE. The "We Card" program: tobacco industry "youth smoking prevention" as industry self-preservation. *Am J Public Health* 2010;100(7):1188-201.

13. Arvey SR, Mahne RE. Advance and retreat: Tobacco control policy in the U.S. military. *Military Medicine* 2008;173(10):985-91.

14. Assunta M, Fields N, Knight J, Chapman S. "Care and feeding": the Asian environmental tobacco smoke consultants programme. *Tob Control* 2004;13 Suppl 2:ii4-12.

15. Assunta M, Chapman S. A mire of highly subjective and ineffective voluntary guidelines: tobacco industry efforts to thwart tobacco control in Malaysia. *Tob Control* 2004;13 Suppl 2:ii43-50.

16. Assunta M, Chapman S. "The world's most hostile environment": how the tobacco industry circumvented Singapore's advertising ban. *Tob Control* 2004;13 Suppl 2:ii51-7.

17. Assunta M, Chapman S. A "clean cigarette" for a clean nation: a case study of Salem Pianissimo in Japan. *Tob Control* 2004;13 Suppl 2:ii58-62.

18. Assunta M, Chapman S. The tobacco industry's accounts of refining indirect tobacco advertising in Malaysia. *Tob Control* 2004;13 Suppl 2:ii63-70.

19. Assunta M, Chapman S. Industry sponsored youth smoking prevention programme in Malaysia: A case study in duplicity. *Tobacco Control* 2004;13(SUPPL. 2):ii37-ii42.

20. Assunta M, Chapman S. The lightest market in the world: light and mild cigarettes in Japan. *Nicotine Tob Res* 2008;10(5):803-10.

21. Baba A, Cook DM, McGarity TO, Bero LA. Legislating "sound science": The role of the tobacco industry. *American Journal of Public Health* 2005;95(SUPPL. 1):S20-S27.

22. Balbach ED, Gasior RJ, Barbeau EM. Tobacco industry documents: comparing the Minnesota Depository and internet access. *Tob Control* 2002;11(1):68-72.

23. Balbach ED, Gasior RJ, Barbeau EM. R.J. Reynolds' targeting of African Americans: 1988-2000. *Am J Public Health* 2003;93(5):822-7.

24. Balbach ED, Barbeau EM, Manteufel V, Pan J. Political coalitions for mutual advantage: the case of the Tobacco Institute's Labor Management Committee. *Am J Public Health* 2005;95(6):985-93.

25. Balbach ED, Herzberg A, Barbeau EM. Political coalitions and working women: how the tobacco industry built a relationship with the Coalition of Labor Union Women. *J Epidemiol Community Health* 2006;60 Suppl 2:27-32.

26. Balbach ED, Campbell RB. Union women, the tobacco industry, and excise taxes: a lesson in unintended consequences. *Am J Prev Med* 2009;37(2 Suppl):S121-5.

27. Barbeau EM, Leavy-Sperounis A, Balbach ED. Smoking, social class, and gender: what can public health learn from the tobacco industry about disparities in smoking? *Tob Control* 2004;13(2):115-20.

28. Barbeau EM, Kelder G, Ahmed S, Mantuefel V, Balbach ED. From strange bedfellows to natural allies: the shifting allegiance of fire service organisations in the push for federal fire-safe cigarette legislation. *Tob Control* 2005;14(5):338-45.

29. Barnes DE, Hanauer P, Slade J, Bero LA, Glantz SA. Environmental tobacco smoke. The Brown and Williamson documents. *JAMA* 1995;274(3):248-53.

30. Barnes RL, Hammond SK, Glantz SA. The tobacco industry's role in the 16 Cities Study of secondhand tobacco smoke: do the data support the stated conclusions? *Environ Health Perspect* 2006;114(12):1890-7.

31. Barnoya J, Glantz S. Tobacco industry success in preventing regulation of secondhand smoke in Latin America: the "Latin Project". *Tob Control* 2002;11(4):305-14.

32. Barnoya J, Glantz SA. The tobacco industry's worldwide ETS consultants project: European and Asian components. *Eur J Public Health* 2006;16(1):69-77.

33. Bero L, Barnes DE, Hanauer P, Slade J, Glantz SA. Lawyer control of the tobacco industry's external research program. The Brown and Williamson documents. *JAMA* 1995;274(3):241-7.

34. Bero LA, Glantz S, Hong MK. The limits of competing interest disclosures. *Tob Control* 2005;14(2):118-26.

35. Bialous SA, Yach D. Whose standard is it, anyway? How the tobacco industry determines the International Organization for Standardization (ISO) standards for tobacco and tobacco products. *Tob Control* 2001;10(2):96-104.

36. Bialous SA, Glantz SA. ASHRAE Standard 62: tobacco industry's influence over national ventilation standards. *Tob Control* 2002;11(4):315-28.

37. Bialous SA, Mochizuki-Kobayashi Y, Stillman F. Courtesy and the challenges of implementing smoke-free policies in Japan. *Nicotine Tob Res* 2006;8(2):203-16.

38. Bialous SA, Presman S, Gigliotti A, Muggli M, Hurt R. [Response of the tobacco industry to the creation of smoke-free environments in Brazil]. *Rev Panam Salud Publica* 2010;27(4):283-90.

39. Bitton A, Neuman MD, Barnoya J, Glantz SA. The p53 tumour suppressor gene and the tobacco industry: research, debate, and conflict of interest. *Lancet* 2005;365(9458):531-40.

40. Bond L, Daube M, Chikritzhs T. Selling addictions: Similarities in approaches between big tobacco and big booze. *Australasian Medical Journal* 2010;3(6):325-32.

41. Bornhauser A, McCarthy J, Glantz SA. German tobacco industry's successful efforts to maintain scientific and political respectability to prevent regulation of secondhand smoke. *Tob Control* 2006;15(2):e1.

42. Braun S, Mejia R, Ling PM, Perez-Stable EJ. Tobacco industry targeting youth in Argentina. *Tob Control* 2008;17(2):111-7.

43. Bryan-Jones K, Bero LA. Tobacco industry efforts to defeat the occupational safety and health administration indoor air quality rule. *Am J Public Health* 2003;93(4):585-92.

44. Burch T, Wander N, Collin J. Uneasy money: the Instituto Carlos Slim de la Salud, tobacco philanthropy and conflict of interest in global health. *Tob Control* 2010;19(6):e1-9.

45. Campbell R, Balbach ED. Mobilising public opinion for the tobacco industry: the Consumer Tax Alliance and excise taxes. *Tob Control* 2008;17(5):351-6.

46. Campbell RB, Balbach ED. Building alliances in unlikely places: progressive allies and the Tobacco Institute's coalition strategy on cigarette excise taxes. *Am J Public Health* 2009;99(7):1188-96.

47. Campbell RB, Balbach ED. Manufacturing credibility: the National Energy Management Institute and the Tobacco Institute's strategy for indoor air quality. *Am J Public Health* 2011;101(3):497-503.

48. Carlini BH, Patrick DL, Halperin AC, Santos V. The tobacco industry's response to the COMMIT Trial: an analysis of legacy tobacco documents. *Public Health Rep* 2006;121(5):501-8.

49. Carlyle J, Collin J, Muggli ME, Hurt RD. British American tobacco and formula one motor racing. *British Medical Journal* 2004;329(7457):104-06.

50. Carpenter CM, Wayne GF, Connolly GN. Designing cigarettes for women: new findings from the tobacco industry documents. *Addiction* 2005;100(6):837-51.

51. Carpenter CM, Wayne GF, Pauly JL, Koh HK, Connolly GN. New cigarette brands with flavors that appeal to youth: tobacco marketing strategies. *Health Aff (Millwood)* 2005;24(6):1601-10.

52. Carpenter CM, Wayne GF, Connolly GN. The role of sensory perception in the development and targeting of tobacco products. *Addiction* 2007;102(1):136-47.

53. Carpenter CM, Connolly GN, Ayo-Yusuf OA, Wayne GF. Developing smokeless tobacco products for smokers: an examination of tobacco industry documents. *Tob Control* 2009;18(1):54-9.

54. Carter SM, Chapman S. Smoking, disease, and obdurate denial: the Australian tobacco industry in the 1980s. *Tob Control* 2003;12 Suppl 3:iii23-30.

55. Carter SM. Cooperation and control: the Tobacco Institute of Australia. *Tob Control* 2003;12 Suppl 3:iii54-60.

56. Carter SM. From legitimate consumers to public relations pawns: the tobacco industry and young Australians. *Tob Control* 2003;12 Suppl 3:iii71-8.

57. Carter SM. The Australian cigarette brand as product, person, and symbol. *Tob Control* 2003;12 Suppl 3:iii79-86.

58. Carter SM. New frontier, new power: the retail environment in Australia's dark market. *Tob Control* 2003;12 Suppl 3:iii95-101.

59. Carter SM. Going below the line: creating transportable brands for Australia's dark market. *Tob Control* 2003;12 Suppl 3:iii87-94.

60. Cataldo JK, Malone RE. False promises: the tobacco industry, "low tar" cigarettes, and older smokers. *J Am Geriatr Soc* 2008;56(9):1716-23.

61. Cataldo JK, Bero LA, Malone RE. "A delicate diplomatic situation": tobacco industry efforts to gain control of the Framingham Study. *J Clin Epidemiol* 2010;63(8):841-53.

62. Chaloupka FJ, Cummings KM, Morley CP, Horan JK. Tax, price and cigarette smoking: evidence from the tobacco documents and implications for tobacco company marketing strategies. *Tob Control* 2002;11 Suppl 1:I62-72.

63. Chapman S. "We are anxious to remain anonymous": the use of third party scientific and medical consultants by the Australian tobacco industry, 1969 to 1979. *Tob Control* 2003;12 Suppl 3:iii31-7.

64. Chapman S, Carter SM, Peters M. "A deep fragrance of academia": the Australian Tobacco Research Foundation. *Tob Control* 2003;12 Suppl 3:iii38-44.

65. Chapman S. "Keep a low profile": pesticide residue, additives, and freon use in Australian tobacco manufacturing. *Tob Control* 2003;12 Suppl 3:iii45-53.

66. Chapman S, Penman A. "Can't stop the boy": Philip Morris' use of Healthy Buildings International to prevent workplace smoking bans in Australia. *Tob Control* 2003;12 Suppl 3:iii107-12.

67. Chapman S, Carter SM. "Avoid health warnings on all tobacco products for just as long as we can": a history of Australian tobacco industry efforts to avoid, delay and dilute health warnings on cigarettes. *Tob Control* 2003;12 Suppl 3:iii13-22.

68. Chu A, Jiang N, Glantz SA. Transnational tobacco industry promotion of the cigarette gifting custom in China. *Tob Control* 2011.

69. Collin J, Lee K, Gilmore AB. Unlocking the corporate documents of British American Tobacco: an invaluable global resource needs radically improved access. *Lancet* 2004;363(9423):1746-7.

70. Connolly GN, Wayne GD, Lymperis D, Doherty MC. How cigarette additives are used to mask environmental tobacco smoke. *Tob Control* 2000;9(3):283-91.

71. Connolly GN, Behm I, Osaki Y, Wayne GF. The impact of menthol cigarettes on smoking initiation among non-smoking young females in Japan. *Int J Environ Res Public Health* 2011;8(1):1-14.

72. Cook BL, Wayne GF, Keithly L, Connolly G. One size does not fit all: how the tobacco industry has altered cigarette design to target consumer groups with specific psychological and psychosocial needs. *Addiction* 2003;98(11):1547-61.

73. Cook DM, Tong EK, Glantz SA, Bero LA. The power of paperwork: how Philip Morris neutralized the medical code for secondhand smoke. *Health Aff (Millwood)* 2005;24(4):994-1004.

74. Cook DM, Bero LA. The politics of smoking in federal buildings: an executive order case study. *Am J Public Health* 2009;99(9):1588-95.

75. Cortese DK, Lewis MJ, Ling PM. Tobacco industry lifestyle magazines targeted to young adults. *J Adolesc Health* 2009;45(3):268-80.

76. Cruz TB, Wright LT, Crawford G. The menthol marketing mix: targeted promotions for focus communities in the United States. *Nicotine Tob Res* 2010;12 Suppl 2:S147-53.

77. Cummings KM, Morley CP, Hyland A. Failed promises of the cigarette industry and its effect on consumer misperceptions about the health risks of smoking. *Tob Control* 2002;11 Suppl 1:I110-7.

78. Cummings KM, Morley CP, Horan JK, Steger C, Leavell NR. Marketing to America's youth: evidence from corporate documents. *Tob Control* 2002;11 Suppl 1:I5-17.

79. Cussen A, McCool J. Tobacco promotion in the Pacific: the current state of tobacco promotion bans and options for accelerating progress. *Asia-Pacific journal of public health / Asia-Pacific Academic Consortium for Public Health* 2011;23(1):70-78.

80. Davis RM. British American Tobacco ghost-wrote reports on tobacco advertising bans by the International Advertising Association and J J Boddewyn. *Tob Control* 2008;17(3):211-4.

81. Dearlove JV, Glantz SA. Boards of Health as venues for clean indoor air policy making. *Am J Public Health* 2002;92(2):257-65.

82. Dearlove JV, Bialous SA, Glantz SA. Tobacco industry manipulation of the hospitality industry to maintain smoking in public places. *Tob Control* 2002;11(2):94-104.

83. Deland K, Lewis K, Taylor AL. Developing a public policy response to the tobacco industry's targeting of women and girls: the role of the WHO Framework Convention on Tobacco Control. *J Am Med Womens Assoc* 2000;55(5):316-9, 21.

84. Diethelm PA, Rielle JC, McKee M. The whole truth and nothing but the truth? The research that Philip Morris did not want you to see. *Lancet* 2005;366(9479):86-92.

85. Difranza J, Clark D, Pollay R. Cigarette package design: opportunities for disease prevention. *Tob Induc Dis* 2003;1(2):97-109.

86. Douglas CE, Davis RM, Beasley JK. Epidemiology of the third wave of tobacco litigation in the United States, 1994-2005. *Tob Control* 2006;15 Suppl 4:iv9-16.

87. Drope J, Chapman S. Tobacco industry efforts at discrediting scientific knowledge of environmental tobacco smoke: a review of internal industry documents. *J Epidemiol Community Health* 2001;55(8):588-94.

88. Drope J, Glantz S. British Columbia capital regional district 100% smokefree bylaw: a successful public health campaign despite industry opposition. *Tob Control* 2003;12(3):264-8.

89. Drope J, Bialous SA, Glantz SA. Tobacco industry efforts to present ventilation as an alternative to smoke-free environments in North America. *Tob Control* 2004;13 Suppl 1:i41-7.

90. Dunsby J, Bero L. A nicotine delivery device without the nicotine? Tobacco industry development of low nicotine cigarettes. *Tob Control* 2004;13(4):362-9.

91. Dyer C. Tobacco company set up network of sympathetic scientists. *BMJ (Clinical research ed.)* 1998;316(7144):1555.

92. Ferris Wayne G, Connolly GN. Application, function, and effects of menthol in cigarettes: a survey of tobacco industry documents. *Nicotine Tob Res* 2004;6 Suppl 1:S43-54.

93. Ferris Wayne G, Connolly GN, Henningfield JE. Brand differences of free-base nicotine delivery in cigarette smoke: the view of the tobacco industry documents. *Tob Control* 2006;15(3):189-98.

94. Fields N, Chapman S. Chasing Ernst L Wynder: 40 years of Philip Morris' efforts to influence a leading scientist. *J Epidemiol Community Health* 2003;57(8):571-8.

95. Flores ML, Barnoya J, Mejia R, Alderete E, Perez-Stable EJ. Litigation in Argentina: challenging the tobacco industry. *Tob Control* 2006;15(2):90-6.

96. Francey N, Chapman S. "Operation Berkshire": the international tobacco companies' conspiracy. *BMJ* 2000;321(7257):371-4.

97. Freeman B, Chapman S, Rimmer M. The case for the plain packaging of tobacco products. *Addiction* 2008;103(4):580-90.

98. Friedman LC, Daynard RA, Banthin CN. How tobacco-friendly science escapes scrutiny in the courtroom. *Am J Public Health* 2005;95 Suppl 1:S16-20.

99. Friedman LC. Tobacco industry use of judicial seminars to influence rulings in products liability litigation. *Tob Control* 2006;15(2):120-4.

100. Friedman LC. Philip Morris's website and television commercials use new language to mislead the public into believing it has changed its stance on smoking and disease. *Tob Control* 2007;16(6):e9.

101. Gardiner PS. The African Americanization of menthol cigarette use in the United States. *Nicotine Tob Res* 2004;6 Suppl 1:S55-65.

102. Garne D, Watson M, Chapman S, Byrne F. Environmental tobacco smoke research published in the journal Indoor and Built Environment and associations with the tobacco industry. *Lancet* 2005;365(9461):804-9.

103. Garten S, Falkner RV. Continual smoking of mentholated cigarettes may mask the early warning symptoms of respiratory disease. *Prev Med* 2003;37(4):291-6.

104. Garten S, Falkner RV. Role of mentholated cigarettes in increased nicotine dependence and greater risk of tobacco-attributable disease. *Prev Med* 2004;38(6):793-8.

105. Gilmore AB, McKee M. Moving East: how the transnational tobacco industry gained entry to the emerging markets of the former Soviet Union-part I: establishing cigarette imports. *Tob Control* 2004;13(2):143-50.

106. Gilmore A, Collin J, Townsend J. Transnational tobacco company influence on tax policy during privatization of a state monopoly: British American Tobacco and Uzbekistan. *Am J Public Health* 2007;97(11):2001-9.

107. Gilmore AB, McKee M, Collin J. The invisible hand: how British American Tobacco precluded competition in Uzbekistan. *Tob Control* 2007;16(4):239-47.

108. Givel MS, Glantz SA. Tobacco lobby political influence on US state legislatures in the 1990s. *Tob Control* 2001;10(2):124-34.

109. Givel M. A comparison of US and Norwegian regulation of coumarin in tobacco products. *Tob Control* 2003;12(4):401-5.

110. Givel M. Oklahoma tobacco policy-making. *J Okla State Med Assoc* 2005;98(3):89-94.

111. Givel M. Philip Morris' FDA gambit: good for public health? *J Public Health Policy* 2005;26(4):450-68.

112. Givel M. Tobacco industry opposition to designating environmental tobacco smoke through E-codes. *J Public Health Policy* 2005;26(1):75-89.

113. Givel M. A comparison of the impact of U.S. and Canadian cigarette pack warning label requirements on tobacco industry profitability and the public health. *Health Policy* 2007;83(2-3):343-52.

114. Glantz SA, Barnes DE, Bero L, Hanauer P, Slade J. Looking through a keyhole at the tobacco industry. The Brown and Williamson documents. *JAMA* 1995;274(3):219-24.

115. Glantz SA, Fox BJ, Lightwood JM. Tobacco litigation. Issues for public health and public policy. *JAMA* 1997;277(9):751-3.

116. Goldberg ME, Davis RM, O'Keefe AM. The role of tobacco advertising and promotion: themes employed in litigation by tobacco industry witnesses. *Tob Control* 2006;15 Suppl 4:iv54-67.

117. Goldman LK, Glantz SA. The passage and initial implementation of Oregon's Measure 44. *Tob Control* 1999;8(3):311-22.

118. Gruning T, Gilmore AB, McKee M. Tobacco industry influence on science and scientists in Germany. *Am J Public Health* 2006;96(1):20-32.

119. Guardino SD, Daynard RA. Tobacco industry lawyers as "disease vectors". *Tob Control* 2007;16(4):224-8.

120. Gundle KR, Dingel MJ, Koenig BA. 'To prove this is the industry's best hope': big tobacco's support of research on the genetics of nicotine addiction. *Addiction* 2010;105(6):974-83.

121. Gunja M, Wayne GF, Landman A, Connolly G, McGuire A. The case for fire safe cigarettes made through industry documents. *Tob Control* 2002;11(4):346-53.

122. Hafez N, Ling PM. How Philip Morris built Marlboro into a global brand for young adults: implications for international tobacco control. *Tob Control* 2005;14(4):262-71.

123. Hafez N, Ling PM. Finding the Kool Mixx: how Brown & Williamson used music marketing to sell cigarettes. *Tob Control* 2006;15(5):359-66.

124. Hammond D, Collishaw NE, Callard C. Secret science: tobacco industry research on smoking behaviour and cigarette toxicity. *Lancet* 2006;367(9512):781-7.

125. Hammond D, Chaiton M, Lee A, Collishaw N. Destroyed documents: uncovering the science that Imperial Tobacco Canada sought to conceal. *CMAJ* 2009;181(10):691-8.

126. Hanauer P, Slade J, Barnes DE, Bero L, Glantz SA. Lawyer control of internal scientific research to protect against products liability lawsuits. The Brown and Williamson documents. *JAMA* 1995;274(3):234-40.

127. Hastings G, MacFadyen L. A day in the life of an advertising man: review of internal documents from the UK tobacco industry's principal advertising agencies. *BMJ* 2000;321(7257):366-71.

128. He P, Takeuchi T, Yano E. Analysis of a tobacco vector and its actions in china: the activities of japan tobacco. *Tob Induc Dis* 2010;8:13.

129. Hendlin Y, Anderson SJ, Glantz SA. 'Acceptable rebellion': marketing hipster aesthetics to sell Camel cigarettes in the US. *Tob Control* 2010;19(3):213-22.

130. Henningfield JE, Rose CA, Zeller M. Tobacco industry litigation position on addiction: continued dependence on past views. *Tob Control* 2006;15 Suppl 4:iv27-36.

131. Hiilamo H. Tobacco industry strategy to undermine tobacco control in Finland. *Tob Control* 2003;12(4):414-23.

132. Hiilamo HT. Tobacco control implications of the first European product liability suit. *Tob Control* 2005;14(1):22-30.

133. Hiilamo H, Hirschhorn N. Tobacco industry documents from outside sources: new perspectives on industry strategies on local levels. *Cent Eur J Public Health* 2006;14(4):175-9.

134. Hiilamo HT. The impact of strategic funding by the tobacco industry of medical expert witnesses appearing for the defence in the Aho Finnish product liability case. *Addiction* 2007;102(6):979-88.

135. Hiilamo H, Kahl U, Lambe M. The Philip Morris Nordic journalist program: strategies, implementation and outcomes. *Health Policy* 2009;89(1):84-96.

136. Hirschhorn N, Bialous SA, Shatenstein S. Philip Morris' new scientific initiative: an analysis. *Tob Control* 2001;10(3):247-52.

137. Hirschhorn N, Bialous SA. Second hand smoke and risk assessment: what was in it for the tobacco industry? *Tob Control* 2001;10(4):375-82.

138. Hirschhorn N. Corporate social responsibility and the tobacco industry: hope or hype? *Tob Control* 2004;13(4):447-53.

139. Hirschhorn N, Bialous SA, Shatenstein S. The Philip Morris External Research Program: results from the first round of projects. *Tob Control* 2006;15(3):267-9.

140. Holden C, Lee K, Gilmore A, Fooks G, Wander N. Trade policy, health, and corporate influence: British American tobacco and China's accession to the World Trade Organization. *Int J Health Serv* 2010;40(3):421-41.

141. Hong MK, Bero LA. Tobacco industry sponsorship of a book and conflict of interest. *Addiction* 2006;101(8):1202-11.

142. Hurt RD, Robertson CR. Prying open the door to the tobacco industry's secrets about nicotine: the Minnesota Tobacco Trial. *JAMA* 1998;280(13):1173-81.

143. Hurt RD, Ebbert JO, Muggli ME, Lockhart NJ, Robertson CR. Open doorway to truth: legacy of the Minnesota tobacco trial. *Mayo Clin Proc* 2009;84(5):446-56.

144. Ibrahim JK, Tsoukalas TH, Glantz SA. Public health foundations and the tobacco industry: lessons from Minnesota. *Tob Control* 2004;13(3):228-36.

145. Ibrahim JK, Glantz SA. Tobacco industry litigation strategies to oppose tobacco control media campaigns. *Tob Control* 2006;15(1):50-8.

146. Iida K, Proctor RN. Learning from Philip Morris: Japan Tobacco's strategies regarding evidence of tobacco health harms as revealed in internal documents from the American tobacco industry. *Lancet* 2004;363(9423):1820-4.

147. Johnson DM, Wine LA, Zack S, Zimmer E, Wang JH, Weitzel-O'Neill PA, et al. Designing a tobacco counter-marketing campaign for African American youth. *Tob Induc Dis* 2008;4:7.

148. Joseph AM, Muggli M, Pearson KC, Lando H. The cigarette manufacturers' efforts to promote tobacco to the U.S. military. *Mil Med* 2005;170(10):874-80.

149. Katz SK, Lavack AM. Tobacco related bar promotions: insights from tobacco industry documents. *Tob Control* 2002;11 Suppl 1:I92-101.

150. Keithly L, Ferris Wayne G, Cullen DM, Connolly GN. Industry research on the use and effects of levulinic acid: a case study in cigarette additives. *Nicotine Tob Res* 2005;7(5):761-71.

151. Kelder GE, Daynard RA. Tobacco litigation as a public health and cancer control strategy. *J Am Med Womens Assoc* 1996;51(1-2):57-62.

152. King W, Carter SM, Borland R, Chapman S, Gray N. The Australian tar derby: the origins and fate of a low tar harm reduction programme. *Tob Control* 2003;12 Suppl 3:iii61-70.

153. Knight J, Chapman S. "Asia is now the priority target for the world anti-tobacco movement": attempts by the tobacco industry to undermine the Asian anti-smoking movement. *Tob Control* 2004;13 Suppl 2:ii30-6.

154. Knight J, Chapman S. "Asian yuppies...are always looking for something new and different": creating a tobacco culture among young Asians. *Tob Control* 2004;13 Suppl 2:ii22-9.

155. Knight J, Chapman S. "A phony way to show sincerity, as we all well know": Tobacco industry lobbying against tobacco control in Hong Kong. *Tobacco Control* 2004;13(SUPPL. 2):ii13-ii21.

156. Kozlowski LT, O'Connor RJ. Cigarette filter ventilation is a defective design because of misleading taste, bigger puffs, and blocked vents. *Tob Control* 2002;11 Suppl 1:I40-50.

157. Kozlowski LT, Dreschel NA, Stellman SD, Wilkenfeld J, Weiss EB, Goldberg ME. An extremely compensatible cigarette by design: documentary evidence on industry awareness and reactions to the Barclay filter design cheating the tar testing system. *Tob Control* 2005;14(1):64-70.

158. Kozlowski LT, Whetzel CA, Stellman SD, O'Connor RJ. Ignoring puff counts: another shortcoming of the Federal Trade Commission cigarette testing programme. *Tob Control* 2008;17 Suppl 1:i6-9.

159. Krasovsky KS. "The lobbying strategy is to keep excise as low as possible" - tobacco industry excise taxation policy in Ukraine. *Tob Induc Dis* 2010;8:10.

160. Kreslake JM, Wayne GF, Connolly GN. The menthol smoker: tobacco industry research on consumer sensory perception of menthol cigarettes and its role in smoking behavior. *Nicotine Tob Res* 2008;10(4):705-15.

161. Kreslake JM, Wayne GF, Alpert HR, Koh HK, Connolly GN. Tobacco industry control of menthol in cigarettes and targeting of adolescents and young adults. *Am J Public Health* 2008;98(9):1685-92.

162. Kreslake JM, Yerger VB. Tobacco industry knowledge of the role of menthol in chemosensory perception of tobacco smoke. *Nicotine Tob Res* 2010;12 Suppl 2:S98-101.

163. Krugman DM, Quinn WH, Sung Y, Morrison M. Understanding the role of cigarette promotion and youth smoking in a changing marketing environment. *J Health Commun* 2005;10(3):261-78.

164. Kummerfeldt CE, Barnoya J, Bero L. Philip Morris involvement in the development of an air quality laboratory in El Salvador. *Tob Control* 2009;18(3):241-4.

165. Kyriakoudes LM. Historians' testimony on "common knowledge" of the risks of tobacco use: a review and analysis of experts testifying on behalf of cigarette manufacturers in civil litigation. *Tob Control* 2006;15 Suppl 4:iv107-16.

166. Kyriss T, Potschke-Langer M, Gruning T. [The German cigarette industry association--obstructing effective tobacco control in Germany]. *Gesundheitswesen* 2008;70(5):315-24.

167. Lambe M, Hallhagen E, Boethius G. [The cynical game of the tobacco industry. Many years' efforts to deny or cover-up the negative effects of tobacco are revealed by the forced publication of internal documents]. *Lakartidningen* 2002;99(24):2756-62.

168. Lambert A, Sargent JD, Glantz SA, Ling PM. How Philip Morris unlocked the Japanese cigarette market: lessons for global tobacco control. *Tob Control* 2004;13(4):379-87.

169. Landman A. Push or be punished: tobacco industry documents reveal aggression against businesses that discourage tobacco use. *Tob Control* 2000;9(3):339-46.

170. Landman A, Ling PM, Glantz SA. Tobacco industry youth smoking prevention programs: protecting the industry and hurting tobacco control. *Am J Public Health* 2002;92(6):917-30.

171. Landman A, Cortese DK, Glantz S. Tobacco industry sociological programs to influence public beliefs about smoking. *Soc Sci Med* 2008;66(4):970-81.

172. Landman A, Glantz SA. Tobacco industry efforts to undermine policy-relevant research. *Am J Public Health* 2009;99(1):45-58.

173. Lavack AM, Toth G. Tobacco point-of-purchase promotion: examining tobacco industry documents. *Tob Control* 2006;15(5):377-84.

174. Lawrence S, Collin J. Competing with kreteks: transnational tobacco companies, globalisation, and Indonesia. *Tob Control* 2004;13 Suppl 2:ii96-103.

175. Lawrence S. British American Tobacco's failure in Turkey. *Tob Control* 2009;18(1):22-8.

176. Lee K, Gilmore AB, Collin J. Breaking and re-entering: British American Tobacco in China 1979-2000. *Tob Control* 2004;13 Suppl 2:ii88-95.

177. Lee K, Collin J. "Key to the future": British American tobacco and cigarette smuggling in China. *PLoS Med* 2006;3(7):e228.

178. Lee K, Carpenter C, Challa C, Lee S, Connolly GN, Koh HK. The strategic targeting of females by transnational tobacco companies in South Korea following trade liberalization. *Global Health* 2009;5:2.

179. LeGresley EM, Muggli ME, Hurt RD. Playing hide-and-seek with the tobacco industry. *Nicotine Tob Res* 2005;7(1):27-40.

180. LeGresley EM, Muggli ME, Hurt RD. Movie moguls: British American Tobacco's covert strategy to promote cigarettes in Eastern Europe. *Eur J Public Health* 2006;16(5):505-8.

181. Legresley E, Lee K, Muggli ME, Patel P, Collin J, Hurt RD. British American Tobacco and the "insidious impact of illicit trade" in cigarettes across Africa. *Tob Control* 2008;17(5):339-46.

182. Liberman J. The tobacco industry and the law: Litigation and legal responsibility. *Cancer Forum* 2004;28(2):80-82.

183. Ling PM, Glantz SA. Why and how the tobacco industry sells cigarettes to young adults: evidence from industry documents. *Am J Public Health* 2002;92(6):908-16.

184. Ling PM, Glantz SA. Tobacco industry research on smoking cessation. Recapturing young adults and other recent quitters. *J Gen Intern Med* 2004;19(5 Pt 1):419-26.

185. Ling PM, Glantz SA. Tobacco industry consumer research on socially acceptable cigarettes. *Tob Control* 2005;14(5):e3.

186. Ling PM, Haber LA, Wedl S. Branding the rodeo: a case study of tobacco sports sponsorship. *Am J Public Health* 2010;100(1):32-41.

187. Lopipero P, Bero LA. Tobacco interests or the public interest: 20 years of industry strategies to undermine airline smoking restrictions. *Tob Control* 2006;15(4):323-32.

188. Lum KL, Polansky JR, Jackler RK, Glantz SA. Signed, sealed and delivered: "big tobacco" in Hollywood, 1927-1951. *Tob Control* 2008;17(5):313-23.

189. Lum KL, Barnes RL, Glantz SA. Enacting tobacco taxes by direct popular vote in the United States: lessons from 20 years of experience. *Tob Control* 2009;18(5):377-86.

190. MacKenzie R, Collin J, Sriwongcharoen K, Muggli ME. "If we can just 'stall' new unfriendly legislations, the scoreboard is already in our favour": transnational tobacco companies and ingredients disclosure in Thailand. *Tob Control* 2004;13 Suppl 2:ii79-87.

191. MacKenzie R, Collin J, Sriwongcharoen K. Thailand--lighting up a dark market: British American tobacco, sports sponsorship and the circumvention of legislation. *J Epidemiol Community Health* 2007;61(1):28-33.

192. Mackenzie R, Collin J. "A good personal scientific relationship": Philip Morris scientists and the Chulabhorn Research Institute, Bangkok. *PLoS Med* 2008;5(12):1737-48.

193. Magzamen S, Glantz SA. The new battleground: California's experience with smoke-free bars. *Am J Public Health* 2001;91(2):245-52.

194. Malone RE. Tobacco industry surveillance of public health groups: the case of STAT (Stop Teenage Addiction to Tobacco) and INFACT (Infant Formula Action Coalition). *Am J Public Health* 2002;92(6):955-60.

195. Malone RE. UCSF's new center for tobacco control research and education finds valuable lessons in the tobacco industry's internal documents. *J Emerg Nurs* 2003;29(1):75-7.

196. Malone RE. Nursing's involvement in tobacco control: historical perspective and vision for the future. *Nurs Res* 2006;55(4 Suppl):S51-7.

197. Mamudu HM, Hammond R, Glantz S. Tobacco industry attempts to counter the World Bank report Curbing the Epidemic and obstruct the WHO framework convention on tobacco control. *Soc Sci Med* 2008;67(11):1690-9.

198. Mamudu HM, Hammond R, Glantz SA. International trade versus public health during the FCTC negotiations, 1999-2003. *Tob Control* 2010.

199. Mandel LL, Glantz SA. Hedging their bets: tobacco and gambling industries work against smoke-free policies. *Tob Control* 2004;13(3):268-76.

200. Mandel LL, Bialous SA, Glantz SA. Avoiding "truth": tobacco industry promotion of life skills training. *J Adolesc Health* 2006;39(6):868-79.

201. Mangurian CV, Bero LA. Lessons learned from the tobacco industry's efforts to prevent the passage of a workplace smoking regulation. *Am J Public Health* 2000;90(12):1926-30.

202. Marian C, O'Connor RJ, Djordjevic MV, Rees VW, Hatsukami DK, Shields PG. Reconciling human smoking behavior and machine smoking patterns: implications for understanding smoking behavior and the impact on laboratory studies. *Cancer Epidemiol Biomarkers Prev* 2009;18(12):3305-20.

203. Mars SG, Ling PM. Meanings & motives. Experts debating tobacco addiction. *Am J Public Health* 2008;98(10):1793-802.

204. McDaniel PA, Malone RE. Understanding Philip Morris's pursuit of US government regulation of tobacco. *Tob Control* 2005;14(3):193-200.

205. McDaniel PA, Solomon G, Malone RE. The tobacco industry and pesticide regulations: case studies from tobacco industry archives. *Environ Health Perspect* 2005;113(12):1659-65.

206. McDaniel PA, Solomon G, Malone RE. The ethics of industry experimentation using employees: the case of taste-testing pesticide-treated tobacco. *Am J Public Health* 2006;96(1):37-46.

207. McDaniel PA, Smith EA, Malone RE. Philip Morris's Project Sunrise: weakening tobacco control by working with it. *Tob Control* 2006;15(3):215-23.

208. McDaniel PA, Malone RE. "I always thought they were all pure tobacco": American smokers' perceptions of "natural" cigarettes and tobacco industry advertising strategies. *Tob Control* 2007;16(6):e7.

209. McDaniel PA, Intinarelli G, Malone RE. Tobacco industry issues management organizations: creating a global corporate network to undermine public health. *Global Health* 2008;4:2.

210. McDaniel PA, Malone RE. Creating the "desired mindset": Philip Morris's efforts to improve its corporate image among women. *Women Health* 2009;49(5):441-74.

211. Megerdichian CL, Rees VW, Wayne GF, Connolly GN. Internal tobacco industry research on olfactory and trigeminal nerve response to nicotine and other smoke components. *Nicotine Tob Res* 2007;9(11):1119-29.

212. Mejia R, Schoj V, Barnoya J, Flores ML, Perez-Stable EJ. Tobacco Industry Strategies to Obstruct the FCTC in Argentina. *CVD Prev Control* 2008;3(4):173-79.

213. Mejia AB, Ling PM. Tobacco industry consumer research on smokeless tobacco users and product development. *Am J Public Health* 2010;100(1):78-87.

214. Mekemson C, Glantz SA. How the tobacco industry built its relationship with Hollywood. *Tob Control* 2002;11 Suppl 1:I81-91.

215. Muggli ME, Forster JL, Hurt RD, Repace JL. The smoke you don't see: uncovering tobacco industry scientific strategies aimed against environmental tobacco smoke policies. *Am J Public Health* 2001;91(9):1419-23.

216. Muggli ME, Pollay RW, Lew R, Joseph AM. Targeting of Asian Americans and Pacific Islanders by the tobacco industry: results from the Minnesota Tobacco Document Depository. *Tob Control* 2002;11(3):201-9.

217. Muggli ME, Hurt RD. Tobacco industry strategies to undermine the 8th World Conference on Tobacco or Health. *Tob Control* 2003;12(2):195-202.

218. Muggli ME, Hurt RD, Blanke DD. Science for hire: a tobacco industry strategy to influence public opinion on secondhand smoke. *Nicotine Tob Res* 2003;5(3):303-14.

219. Muggli ME, Hurt RD, Repace J. The tobacco industry's political efforts to derail the EPA report on ETS. *Am J Prev Med* 2004;26(2):167-77.

220. Muggli ME, LeGresley EM, Hurt RD. Big tobacco is watching: British American Tobacco's surveillance and information concealment at the Guildford depository. *Lancet* 2004;363(9423):1812-9.

221. Muggli ME, Hurt RD. A cigarette manufacturer and a managed care company collaborate to censor health information targeted at employees. *Am J Public Health* 2004;94(8):1307-11.

222. Muggli ME, Hurt RD, Becker LB. Turning free speech into corporate speech: Philip Morris' efforts to influence U.S. and European journalists regarding the U.S. EPA report on secondhand smoke. *Prev Med* 2004;39(3):568-80.

223. Muggli ME, Ebbert JO, Robertson C, Hurt RD. Waking a sleeping giant: the tobacco industry's response to the polonium-210 issue. *Am J Public Health* 2008;98(9):1643-50.

224. Muggli ME, Lee K, Gan Q, Ebbert JO, Hurt RD. "Efforts to Reprioritise the Agenda" in China: British American Tobacco's Efforts to Influence Public Policy on Secondhand Smoke in China. *PLoS Med* 2008;5(12):1729-69.

225. Muggli ME, Lockhart NJ, Ebbert JO, Jimenez-Ruiz CA, Riesco Miranda JA, Hurt RD. Legislating tolerance: Spain's national public smoking law. *Tob Control* 2010;19(1):24-30.

226. Nakkash R, Lee K. Smuggling as the "key to a combined market": British American Tobacco in Lebanon. *Tob Control* 2008;17(5):324-31.

227. Nakkash R, Lee K. The tobacco industry's thwarting of marketing restrictions and health warnings in Lebanon. *Tob Control* 2009;18(4):310-6.

228. Neilsen K, Glantz SA. A tobacco industry study of airline cabin air quality: Dropping inconvenient findings. *Tobacco Control* 2004;13(SUPPL. 1):i20-i29.

229. Neuman M, Bitton A, Glantz S. Tobacco industry strategies for influencing European Community tobacco advertising legislation. *Lancet* 2002;359(9314):1323-30.

230. Neuman MD, Bitton A, Glantz SA. Tobacco industry influence on the definition of tobacco related disorders by the American Psychiatric Association. *Tob Control* 2005;14(5):328-37.

231. Nixon ML, Mahmoud L, Glantz SA. Tobacco industry litigation to deter local public health ordinances: the industry usually loses in court. *Tob Control* 2004;13(1):65-73.

232. O'Connor RJ, Hurley PJ. Existing technologies to reduce specific toxicant emissions in cigarette smoke. *Tob Control* 2008;17 Suppl 1:i39-48.

233. Offen N, Smith EA, Malone RE. From adversary to target market: the ACT-UP boycott of Philip Morris. *Tob Control* 2003;12(2):203-7.

234. Offen N, Smith EA, Malone RE. The perimetric boycott: a tool for tobacco control advocacy. *Tob Control* 2005;14(4):272-7.

235. Offen N, Smith EA, Malone RE. "Willful misconduct": how the US government prevented tobacco-disabled veterans from obtaining disability pensions. *Am J Public Health* 2010;100(7):1166-73.

236. Ong EK, Glantz SA. Tobacco industry efforts subverting International Agency for Research on Cancer's second-hand smoke study. *Lancet* 2000;355(9211):1253-9.

237. O'Sullivan B, Chapman S. Eyes on the prize: transnational tobacco companies in China 1976-1997. *Tob Control* 2000;9(3):292-302.

238. Otanez MG, Muggli ME, Hurt RD, Glantz SA. Eliminating child labour in Malawi: a British American Tobacco corporate responsibility project to sidestep tobacco labour exploitation. *Tob Control* 2006;15(3):224-30.

239. Otanez MG, Mamudu H, Glantz SA. Global leaf companies control the tobacco market in Malawi. *Tob Control* 2007;16(4):261-9.

240. Otanez MG, Mamudu HM, Glantz SA. Tobacco companies' use of developing countries' economic reliance on tobacco to lobby against global tobacco control: the case of Malawi. *Am J Public Health* 2009;99(10):1759-71.

241. Pan J, Barbeau EM, Levenstein C, Balbach ED. Smoke-free airlines and the role of organized labor: a case study. *Am J Public Health* 2005;95(3):398-404.

242. Panzano VC, Wayne GF, Pickworth WB, Connolly GN. Human electroencephalography and the tobacco industry: a review of internal documents. *Tob Control* 2010;19(2):153-9.

243. Patel P, Collin J, Gilmore AB. "The law was actually drafted by us but the Government is to be congratulated on its wise actions": British American Tobacco and public policy in Kenya. *Tob Control* 2007;16(1):e1.

244. Pauly JL, Mepani AB, Lesses JD, Cummings KM, Streck RJ. Cigarettes with defective filters marketed for 40 years: what Philip Morris never told smokers. *Tob Control* 2002;11 Suppl 1:I51-61.

245. Pauly JL, O'Connor RJ, Paszkiewicz GM, Cummings KM, Djordjevic MV, Shields PG. Cigarette filter-based assays as proxies for toxicant exposure and smoking behavior--a literature review. *Cancer Epidemiol Biomarkers Prev* 2009;18(12):3321-33.

246. Perez-Martin J, Peruga A. [The International Association of Tobacco Growers attacks WHO]. *Rev Panam Salud Publica* 2003;13(4):267-70.

247. Perry CL. The tobacco industry and underage youth smoking: tobacco industry documents from the Minnesota litigation. *Arch Pediatr Adolesc Med* 1999;153(9):935-41.

248. Petticrew MP, Lee K. The "Father of Stress" Meets "Big Tobacco": Hans Selye and the Tobacco Industry. *Am J Public Health* 2010.

249. Pilkington P, Gilmore AB. The Living Tomorrow Project: how Philip Morris has used a Belgian tourist attraction to promote ventilation approaches to the control of second hand smoke. *Tob Control* 2004;13(4):375-8.

250. Pion M, Givel MS. Airport smoking rooms don't work. *Tobacco Control* 2004;13(SUPPL. 1):i37-i40.

251. Pollay RW. Targeting youth and concerned smokers: evidence from Canadian tobacco industry documents. *Tob Control* 2000;9(2):136-47.

252. Pollay RW, Dewhirst T. The dark side of marketing seemingly "Light" cigarettes: successful images and failed fact. *Tob Control* 2002;11 Suppl 1:I18-31.

253. Pollay RW, Dewhirst T. A Premiere example of the illusion of harm reduction cigarettes in the 1990s. *Tob Control* 2003;12(3):322-32.

254. Pollock D. Forty years on: a war to recognise and win. How the tobacco industry has survived the revelations on smoking and health. *Br Med Bull* 1996;52(1):174-82.

255. Prochaska JJ, Hall SM, Bero LA. Tobacco use among individuals with schizophrenia: what role has the tobacco industry played? *Schizophr Bull* 2008;34(3):555-67.

256. Proctor RN. Should medical historians be working for the tobacco industry? *Lancet* 2004;363(9416):1174-75.

257. Rabinoff M, Caskey N, Rissling A, Park C. Pharmacological and chemical effects of cigarette additives. *Am J Public Health* 2007;97(11):1981-91.

258. Raebeck A, Campbell R, Balbach E. Unhealthy partnerships: the tobacco industry and African American and Latino labor organizations. *J Immigr Minor Health* 2010;12(2):228-33.

259. Rees VW, Kreslake JM, O'Connor RJ, Cummings KM, Parascandola M, Hatsukami D, et al. Methods used in internal industry clinical trials to assess tobacco risk reduction. *Cancer Epidemiol Biomarkers Prev* 2009;18(12):3196-208.

260. Rees VW, Kreslake JM, Cummings KM, O'Connor RJ, Hatsukami DK, Parascandola M, et al. Assessing consumer responses to potential reduced-exposure tobacco products: a review of tobacco industry and independent research methods. *Cancer Epidemiol Biomarkers Prev* 2009;18(12):3225-40.

261. Rego B. The Polonium brief: a hidden history of cancer, radiation, and the tobacco industry. *Isis* 2009;100(3):453-84.

262. Ritch WA, Begay ME. Smoke and mirrors: how Massachusetts diverted millions in tobacco tax revenues. *Tob Control* 2001;10(4):309-16.

263. Ritch WA, Begay ME. Strange bedfellows: the history of collaboration between the Massachusetts Restaurant Association and the tobacco industry. *Am J Public Health* 2001;91(4):598-603.

264. Ritch WA, Begay ME. Smoke and mirrors: how Massachusetts diverted millions in tobacco tax revenues. *J Epidemiol Community Health* 2002;56(7):522-8.

265. Saloojee Y, Dagli E. Tobacco industry tactics for resisting public policy on health. *Bull World Health Organ* 2000;78(7):902-10.

266. Schane RE, Glantz SA, Ling PM. Social smoking implications for public health, clinical practice, and intervention research. *Am J Prev Med* 2009;37(2):124-31.

267. Schick S, Glantz S. Scientific analysis of second-hand smoke by the tobacco industry, 1929-1972. *Nicotine Tob Res* 2005;7(4):591-612.

268. Schick S, Glantz S. Philip Morris toxicological experiments with fresh sidestream smoke: more toxic than mainstream smoke. *Tob Control* 2005;14(6):396-404.

269. Schick SF, Glantz S. Concentrations of the carcinogen 4-(methylnitrosamino)-1-(3-pyridyl)-1-butanone in sidestream cigarette smoke increase after release into indoor air: results from unpublished tobacco industry research. *Cancer Epidemiol Biomarkers Prev* 2007;16(8):1547-53.

270. Schick Stanton A Glantz SF. Old ways, new means: Tobacco industry funding of academic and private sector scientists since the Master Settlement Agreement. *Tobacco Control* 2007;16(3):157-64.

271. Sebrie EM, Barnoya J, Perez-Stable EJ, Glantz SA. Tobacco industry successfully prevented tobacco control legislation in Argentina. *Tob Control* 2005;14(5):e2.

272. Sebrie EM, Glantz SA. Attempts to undermine tobacco control: tobacco industry "youth smoking prevention" programs to undermine meaningful tobacco control in Latin America. *Am J Public Health* 2007;97(8):1357-67.

273. Sebrie EM, Glantz SA. "Accommodating" smoke-free policies: tobacco industry's Courtesy of Choice programme in Latin America. *Tob Control* 2007;16(5):e6.

274. Shafey O, Fernandez E, Thun M, Schiaffino A, Dolwick S, Cokkinides V. Cigarette advertising and female smoking prevalence in Spain, 1982-1997: case studies in International Tobacco Surveillance. *Cancer* 2004;100(8):1744-9.

275. Shamasunder B, Bero L. Financial ties and conflicts of interest between pharmaceutical and tobacco companies. *JAMA* 2002;288(6):738-44.

276. Slade J, Bero LA, Hanauer P, Barnes DE, Glantz SA. Nicotine and addiction. The Brown and Williamson documents. *JAMA* 1995;274(3):225-33.

277. Smith EA, Malone RE. Altria means tobacco: Philip Morris's identity crisis. *Am J Public Health* 2003;93(4):553-6.

278. Smith EA, Malone RE. The outing of Philip Morris: advertising tobacco to gay men. *Am J Public Health* 2003;93(6):988-93.

279. Smith EA, Malone RE. Thinking the "unthinkable": why Philip Morris considered quitting. *Tob Control* 2003;12(2):208-13.

280. Smith EA. 'It's interesting how few people die from smoking': tobacco industry efforts to minimize risk and discredit health promotion. *Eur J Public Health* 2007;17(2):162-70.

281. Smith EA, Blackman VS, Malone RE. Death at a discount: how the tobacco industry thwarted tobacco control policies in US military commissaries. *Tob Control* 2007;16(1):38-46.

282. Smith EA, Malone RE. 'We will speak as the smoker': the tobacco industry's smokers' rights groups. *Eur J Public Health* 2007;17(3):306-13.

283. Smith EA, Malone RE. Philip Morris's health information web site appears responsible but undermines public health. *Public Health Nurs* 2008;25(6):554-64.

284. Smith EA, Malone RE. "Everywhere the soldier will be": wartime tobacco promotion in the US military. *Am J Public Health* 2009;99(9):1595-602.

285. Smith EA, Malone RE. Tobacco promotion to military personnel: "the plums are here to be plucked". *Mil Med* 2009;174(8):797-806.

286. Smith KE, Fooks G, Collin J, Weishaar H, Mandal S, Gilmore AB. "Working the system"--British American tobacco's influence on the European union treaty and its implications for policy: an analysis of internal tobacco industry documents. *PLoS Med* 2010;7(1):e1000202.

287. Smith EA, McDaniel PA. Covering their butts: responses to the cigarette litter problem. *Tob Control* 2010.

288. Soto-Mas F, Villalbi JR, Granero L, Jacobson H, Balcazar H. [The tobacco industry's internal documents and smoking prevention in Spain]. *Gac Sanit* 2003;17 Suppl 3:9-14.

289. Stanton CR, Chu A, Collin J, Glantz SA. Promoting tobacco through the international language of dance music: British American Tobacco and the Ministry of Sound. *Eur J Public Health* 2010.

290. Stevens P, Carlson LM, Hinman JM. An analysis of tobacco industry marketing to lesbian, gay, bisexual, and transgender (LGBT) populations: strategies for mainstream tobacco control and prevention. *Health Promot Pract* 2004;5(3 Suppl):129S-34S.

291. Sullivan S, Glantz S. The changing role of agriculture in tobacco control policymaking: a South Carolina case study. *Soc Sci Med* 2010;71(8):1527-34.

292. Szczypka G, Wakefield MA, Emery S, Terry-McElrath YM, Flay BR, Chaloupka FJ. Working to make an image: an analysis of three Philip Morris corporate image media campaigns. *Tob Control* 2007;16(5):344-50.

293. Szilagyi T, Chapman S. Hungry for Hungary: examples of tobacco industry's expansionism. *Cent Eur J Public Health* 2003;11(1):38-43.

294. Szilagyi T, Chapman S. Tobacco industry efforts to keep cigarettes affordable: a case study from Hungary. *Cent Eur J Public Health* 2003;11(4):223-8.

295. Szilagyi T, Chapman S. Tobacco industry efforts to erode tobacco advertising controls in Hungary. *Cent Eur J Public Health* 2004;12(4):190-6.

296. Tesler LE, Malone RE. Corporate philanthropy, lobbying, and public health policy. *Am J Public Health* 2008;98(12):2123-33.

297. Thomson G, Wilson N. Implementation failures in the use of two New Zealand laws to control the tobacco industry: 1989-2005. *Aust New Zealand Health Policy* 2005;2:32.

298. Toll BA, Ling PM. The Virginia Slims identity crisis: an inside look at tobacco industry marketing to women. *Tob Control* 2005;14(3):172-80.

299. Tong EK, Glantz SA. ARTIST (Asian regional tobacco industry scientist team): Philip Morris' attempt to exert a scientific and regulatory agenda on Asia. *Tob Control* 2004;13 Suppl 2:ii118-24.

300. Tong EK, England L, Glantz SA. Changing conclusions on secondhand smoke in a sudden infant death syndrome review funded by the tobacco industry. *Pediatrics* 2005;115(3):e356-66.

301. Tong EK, Glantz SA. Tobacco industry efforts undermining evidence linking secondhand smoke with cardiovascular disease. *Circulation* 2007;116(16):1845-54.

302. Trotter L, Chapman S. "Conclusions about exposure to ETS and health that will be unhelpful to us": how the tobacco industry attempted to delay and discredit the 1997 Australian National Health and Medical Research Council report on passive smoking. *Tob Control* 2003;12 Suppl 3:iii102-6.

303. Tsoukalas TH, Glantz SA. Development and destruction of the first state funded anti-smoking campaign in the USA. *Tob Control* 2003;12(2):214-20.

304. Wakefield M, Morley C, Horan JK, Cummings KM. The cigarette pack as image: new evidence from tobacco industry documents. *Tob Control* 2002;11 Suppl 1:I73-80.

305. Wakefield M, McLeod K, Perry CL. "Stay away from them until you're old enough to make a decision": tobacco company testimony about youth smoking initiation. *Tob Control* 2006;15 Suppl 4:iv44-53.

306. Wander N, Malone RE. Selling off or selling out? Medical schools and ethical leadership in tobacco stock divestment. *Acad Med* 2004;79(11):1017-26.

307. Wander N, Malone RE. Fiscal versus social responsibility: how Philip Morris shaped the public funds divestment debate. *Tob Control* 2006;15(3):231-41.

308. Wander N, Malone RE. Making big tobacco give in: you lose, they win. *Am J Public Health* 2006;96(11):2048-54.

309. Washington HA. Burning Love: big tobacco takes aim at LGBT youths. *Am J Public Health* 2002;92(7):1086-95.

310. Wayne GF, Connolly GN. How cigarette design can affect youth initiation into smoking: Camel cigarettes 1983-93. *Tob Control* 2002;11 Suppl 1:I32-9.

311. Wayne GF, Connolly GN, Henningfield JE. Assessing internal tobacco industry knowledge of the neurobiology of tobacco dependence. *Nicotine Tob Res* 2004;6(6):927-40.

312. Wayne GF, Connolly GN, Henningfield JE, Farone WA. Tobacco industry research and efforts to manipulate smoke particle size: implications for product regulation. *Nicotine Tob Res* 2008;10(4):613-25.

313. Wayne GF, Connolly GN. Regulatory assessment of brand changes in the commercial tobacco product market. *Tob Control* 2009;18(4):302-9.

314. Wayne GF, Carpenter CM. Tobacco industry manipulation of nicotine dosing, 2009:457-85.

315. Wen CP, Chen T, Tsai YY, Tsai SP, Chung WS, Cheng TY, et al. Are marketing campaigns in Taiwan by foreign tobacco companies targeting young smokers? *Tob Control* 2005;14 Suppl 1:i38-44.

316. Wen CP, Cheng TY, Eriksen MP, Tsai SP, Hsu CC. The impact of the cigarette market opening in Taiwan. *Tob Control* 2005;14 Suppl 1:i4-9.

317. Wen CP, Peterson RA, Cheng TY, Tsai SP, Eriksen MP, Chen T. Paradoxical increase in cigarette smuggling after the market opening in Taiwan. *Tob Control* 2006;15(3):160-5.

318. White J, Bero LA. Public health under attack: the American Stop Smoking Intervention Study (ASSIST) and the tobacco industry. *Am J Public Health* 2004;94(2):240-50.

319. White J, Parascandola M, Bero L. Tobacco industry research and protection of human subjects: a case study of R. J. Reynolds. *Nicotine Tob Res* 2007;9(11):1213-25.

320. Willems EW, Rambali B, Vleeming W, Opperhuizen A, van Amsterdam JG. Significance of ammonium compounds on nicotine exposure to cigarette smokers. *Food Chem Toxicol* 2006;44(5):678-88.

321. Yerger VB, Malone RE. African American leadership groups: smoking with the enemy. *Tob Control* 2002;11(4):336-45.

322. Yerger VB, Daniel MR, Malone RE. Taking it to the streets: responses of African American young adults to internal tobacco industry documents. *Nicotine Tob Res* 2005;7(1):163-72.

323. Yerger VB, Malone RE. Melanin and nicotine: A review of the literature. *Nicotine Tob Res* 2006;8(4):487-98.

324. Yerger VB, Przewoznik J, Malone RE. Racialized geography, corporate activity, and health disparities: tobacco industry targeting of inner cities. *J Health Care Poor Underserved* 2007;18(4 Suppl):10-38.

325. Zhong F, Yano E. British American Tobacco's tactics during China's accession to the World Trade Organization. *Tob Control* 2007;16(2):133-7.

II. Articles using pharmaceutical company documents (n=20)

IIA. Articles retrieved from original search (n=16)

1. Applbaum K. Getting to yes: corporate power and the creation of a psychopharmaceutical blockbuster. *Cult Med Psychiatry* 2009;33(2):185-215.

2. Bernschneider-Reif S, Oxler F, Freudenmann RW. The origin of MDMA ("ecstasy")--separating the facts from the myth. *Pharmazie* 2006;61(11):966-72.

3. Breggin PR. Court filing makes public my previously suppressed analysis of Paxil's effects. *Ethical Hum Psychol Psychiatry* 2006;8(1):77-84.

4. Fugh-Berman AJ. The haunting of medical journals: how ghostwriting sold "HRT". *PLoS Med* 2010;7(9):e1000335.

5. Greene JA. Releasing the flood waters: diuril and the reshaping of hypertension. *Bull Hist Med* 2005;79(4):749-94.

6. Hill KP, Ross JS, Egilman DS, Krumholz HM. The ADVANTAGE seeding trial: a review of internal documents. *Ann Intern Med* 2008;149(4):251-8.

7. Jureidini JN, McHenry LB, Mansfield PR. Clinical trials and drug promotion: Selective reporting of study 329. *International Journal of Risk and Safety in Medicine* 2008;20(1-2):73-81.

8. McHenry LB, Jureidini JN. Industry-sponsored ghostwriting in clinical trial reporting: a case study. *Account Res* 2008;15(3):152-67.

9. Psaty BM, Furberg CD, Ray WA, Weiss NS. Potential for conflict of interest in the evaluation of suspected adverse drug reactions: use of cerivastatin and risk of rhabdomyolysis. *JAMA* 2004;292(21):2622-31.

10. Psaty BM, Kronmal RA. Reporting mortality findings in trials of rofecoxib for Alzheimer disease or cognitive impairment: a case study based on documents from rofecoxib litigation. *JAMA* 2008;299(15):1813-7.

11. Ross JS, Hill KP, Egilman DS, Krumholz HM. Guest authorship and ghostwriting in publications related to rofecoxib: a case study of industry documents from rofecoxib litigation. *JAMA* 2008;299(15):1800-12.

12. Spielmans GI. The promotion of olanzapine in primary care: an examination of internal industry documents. *Soc Sci Med* 2009;69(1):14-20.

13. Steinman MA, Bero LA, Chren MM, Landefeld CS. Narrative review: the promotion of gabapentin: an analysis of internal industry documents. *Ann Intern Med* 2006;145(4):284-93.

14. Steinman MA, Harper GM, Chren MM, Landefeld CS, Bero LA. Characteristics and impact of drug detailing for gabapentin. *PLoS Med* 2007;4(4):e134.

15. Vedula SS, Bero L, Scherer RW, Dickersin K. Outcome reporting in industry-sponsored trials of gabapentin for off-label use. *N Engl J Med* 2009;361(20):1963-71.

16. Woods S, Tek C, Srihari VH. Adverse drug effects not detected at licensing review: Regulatory autopsy of olanzapine. *Schizophrenia Bulletin* 2011;37:30.

IIB. Articles retrieved through searches of other sources (n=4)

1. Jureidini JN, McHenry LB. Conflicted medical journals and the failure of trust. *Account Res* 2011;18(1):45-54.

2. Krumholz HM, Ross JS, Presler AH, Egilman DS. What have we learnt from Vioxx? *BMJ* 2007;334(7585):120-3.

3. Landefeld CS, Steinman MA. The Neurontin legacy--marketing through misinformation and manipulation. *N Engl J Med* 2009;360(2):103-6.

4. Ross JS, Madigan D, Konstam MA, Egilman DS, Krumholz HM. Persistence of cardiovascular risk after rofecoxib discontinuation. *Arch Intern Med* 2010;170(22):2035-6.

III. Articles not using tobacco or pharmaceutical company documents (n=16)*

*Egilman 2003 used documents from both mining and manufacturing companies.

1. Boice JD, Jr., Marano DE, Fryzek JP, Sadler CJ, McLaughlin JK. Mortality among aircraft manufacturing workers. *Occup Environ Med* 1999;56(9):581-97.

2. Castleman B. Asbestos products, hazards, and regulation. *Int J Health Serv* 2006;36(2):295-307.

3. Clapp RW. Mortality among US employees of a large computer manufacturing company: 1969-2001. *Environ Health* 2006;5:30.

4. Egilman D, Wallace W, Hom C. Corporate corruption of medical literature: asbestos studies concealed by W.R. Grace & Co. *Account Res* 1998;6(1-2):127-47.

5. Egilman D, Bagley S, Biklen M, Golub AS, Bohme SR. The beryllium "double standard" standard. *Int J Health Serv* 2003;33(4):769-812.

6. Egilman D, Mailloux C, Valentin C. Popcorn-worker lung caused by corporate and regulatory negligence: an avoidable tragedy. *Int J Occup Environ Health* 2007;13(1):85-98.

7. Greenberg M. Cape Asbestos, Barking, health and environment: 1928-1946. *Am J Ind Med* 2003;43(2):109-19.

8. Greenberg M. Biological effects of asbestos: New York Academy of Sciences 1964. *Am J Ind Med* 2003;43(5):543-52.

9. Johnson CJ. Environmental and health effects of the nuclear industry and nuclear weapons: a current evaluation. *Ecol Dis* 1982;1(2-3):135-52.

10. Lilienfeld DE. The silence: the asbestos industry and early occupational cancer research--a case study. *Am J Public Health* 1991;81(6):791-800.

11. Pelfrene A. Glyphosate: Toxicology and Human Risk Assessment. *Environnement, Risques et Sante* 2003;2(6):323-34.

12. Robinson JC. Consolidation of medical groups into physician practice management organizations. *JAMA* 1998;279(2):144-9.

13. Rosen S, Vincent JR, MacLeod W, Fox M, Thea DM, Simon JL. The cost of HIV/AIDS to businesses in southern Africa. *AIDS* 2004;18(2):317-24.

14. Rotarius T, Liberman A, Trujillo A. Economic contributions of physicians--the financial impact on their community. *Health Care Manag (Frederick)* 2008;27(4):317-23.

15. Steenland K, Pinkerton LE. Mortality patterns following downsizing at Pan American World Airways. *Am J Epidemiol* 2008;167(1):1-6.

16. Wright EJ, Haslam RA. Manual handling risks and controls in a soft drinks distribution centre. *Appl Ergon* 1999;30(4):311-8.
